# Sex- and time-dependent role of insulin regulated aminopeptidase in lipopolysaccharide-induced inflammation

**DOI:** 10.3389/fimmu.2024.1466692

**Published:** 2024-10-04

**Authors:** Anika Vear, Amlan Chakraborty, Farnaz Fahimi, Dorota Ferens, Robert Widdop, Chrishan S. Samuel, Tracey Gaspari, Peter M. van Endert, Siew Yeen Chai

**Affiliations:** ^1^ Department of Physiology, Biomedicine Discovery Institute, Monash University, Clayton, VIC, Australia; ^2^ Novo Nordisk Foundation Centre for Basic Metabolic Research, University of Copenhagen, Copenhagen, Denmark; ^3^ Department of Pharmacology, Biomedicine Discovery Institute, Monash University, Clayton, VIC, Australia; ^4^ Division of Immunology, Immunity to Infection and Respiratory Medicine, School of Biological Sciences, The University of Manchester, Manchester, United Kingdom; ^5^ Institut Necker Enfants Malades, Université Paris Cité, INSERM, CNRS, Paris, France; ^6^ Service Immunologie Biologique, Assistance Publique-Hôpitaux de Paris (AP-HP), Hôpital Universitaire Necker-Enfants Malades, Paris, France

**Keywords:** insulin regulated aminopeptidase, IRAP, lipopolysaccharide, LPS, inflammation, gram-negative sepsis

## Abstract

The enzyme, insulin regulated aminopeptidase (IRAP), is expressed in multiple immune cells such as macrophages, dendritic cells and T cells, where it plays a role in regulating the innate and adaptive immune response. There is a genetic association between IRAP and survival outcomes in patients with septic shock where a variant of its gene was found to be associated with increased 28-day mortality. This study investigated the role for IRAP in a lipopolysaccharide (LPS)-induced inflammatory response which is thought to model facets of the systemic inflammation observed in the early stages of human gram-negative sepsis. The frequencies and activation of splenic immune cell populations were investigated in the IRAP knockout (KO) mice compared to the wildtype controls over a period of 4-, 24-, or 48-hours following LPS stimulation. Dendritic cells isolated from the spleen of female IRAP KO mice, displayed significant increases in the activation markers CD40, CD86 and MHCII at 24 hours after LPS induction. A modest heightened pro-inflammatory response to LPS was observed with increased expression of activation marker CD40 in M1 macrophages from male IRAP knockout mice. Observations *in vitro* in bone marrow-derived macrophages (BMDM) revealed a heightened pro-inflammatory response to LPS with significant increases in the expression of CD40 in IRAP deficient cells compared with BMDM from WT mice. The heightened LPS-induced response was associated with increased pro-inflammatory cytokine secretion in these BMDM cells. A genotype difference was also detected in the BMDM from female mice displaying suppression of the LPS-induced increases in the activation markers CD40, CD86, CD80 and MHCII in IRAP deficient cells. Thus, this study suggests that IRAP plays specific time- and sex-dependent roles in the LPS-induced inflammatory response in dendritic cells and macrophages.

## Introduction

Sepsis is a life-threatening condition in which there is dysregulation of the host’s response to an infection (1). It is estimated that globally 31.5 million patients develop sepsis each year, placing a significant burden on healthcare systems with patients requiring intensive care support as they are at high risk of developing complications such as acute kidney injury and acute respiratory distress syndrome (2). The pathogenesis of sepsis is multifaceted and can be divided into 3 stages, starting with an acute systemic inflammatory response, then dysregulation of hemostasis, and finally immunosuppression and the progression to septic shock that can result in organ damage and death (3). Sepsis is the leading cause of death in intensive care units and initial treatment options were focused on improving 30-day survival by dampening of the inflammatory response (4, 5).

Insulin regulated aminopeptidase (IRAP) is a transmembrane enzyme which regulates the processing of peptide hormones such as arginine-vasopressin (AVP) and oxytocin, as well as peptide trimming for major histocompatibility complex (MHC) I cross presentation. It has been implicated to play a role in sepsis with a single nucleotide polymorphism (SNP) variant of the IRAP gene (rs18059) associated with an increase in 28-day mortality in patients with septic shock (6). Furthermore, this IRAP polymorphism was associated with increased plasma AVP clearance which is physiologically relevant as this vasoactive peptide participates in the regulation of cardiovascular homeostasis in hypotensive states during septic shock (6).

IRAP is highly expressed in several immune cells including dendritic cells (DCs) (7), macrophages (8), mast cells (9), and T cells (10) and is thought to regulate facets of both innate and adaptive immune responses. For example, the N-terminal cytosolic domain of IRAP has been implicated in the regulation of the intracellular trafficking of endosomes containing the receptor for DNA containing the dinucleotide CpG, TLR9 (7), and the high affinity IgG receptor, FcγRI (11), both in DCs, and the CD3ζ component of the T cell receptor (TCR) complex in T cells (10). The catalytic site in the C-terminal domain of IRAP in the luminal compartment in endosomes participates in the trimming of antigens for cross presentation on MHCI molecules in DCs (12). In alveolar macrophages from neonatal IRAP knockout (KO) mice, there is an increased production of interferon (IFN)-I 24 hours following treatment with respiratory syncytial virus (RSV) compared to cells from wildtype (WT) mice (8). This culminated in the more efficient elimination of the virus in the lungs of newborn IRAP KO mice (8). These findings support previous studies in DCs (7) and T cells (10) where IRAP gene deletion resulted in a heightened pro-inflammatory response following an acute immune challenge. It is worth noting that these studies have examined IRAP function at a single time point and often in only male animals.

Infection of the bacterial endotoxin, lipopolysaccharide (LPS), represents one of three categories of preclinical models of sepsis which have been widely used (13). These models have contributed significant insights into the host defense mechanisms triggered by infections despite limitations including accelerated timing of disease onset and progression compared to the human condition. Although LPS administration may not be regarded as a clinically relevant model as it does not reproduce the hemodynamic changes observed in human sepsis, it induces systemic inflammation that mimics the early clinical features of the condition (13).

The current study investigated potential roles of IRAP on regulating the acute inflammatory response induced by systemic administration of LPS. Multiple immune cell populations at three distinct time points in both male and female mice were examined. Global IRAP gene deletion resulted in time- and sex-dependent changes in the activation state of DCs and macrophages isolated from the spleen, a finding that was recapitulated *in vitro* in bone marrow-derived macrophages isolated from IRAP KO mice.

## Materials and methods

### Materials

The LPS used for treatments in both the *in vivo* and *in vitro* models was from Invivogen (#LPS-EK). M-CSF was purchased from StemCell (#78059.1). Antibodies used for flow cytometry were purchased from BD Bioscience: BV650-CD80, PerCP-Cy5.5-Gr1, PE-CD86, PE-CF594-CD40, BV786-CD45, BUV395-CD11c, PerCP-Cy5.5-CD11b, FITC-CD19, BUV496-CD4, BV421-CD206, APC-TLR4, purified CD16/CD32, or BioLegend: PE-Cy7-MHCII, APC fire 750-F4/80, AF647-CD206, zombie aqua. Antibodies used for immunocytochemistry experiments include BV786-CD45 (BD Bioscience #564225), TNF-α (Abcam #ab6671), IL-1β (Abcam #ab205924), IRAP (Cell Signaling #6918) and TLR4 (Abcam #ab13556). DuoSet ELISA kits were purchased from R&D Systems (TNF-α #DY410, IL-1β #DY401, IL-6 #DY406).

### Animals

WT and global IRAP KO (14) C57BL/6J male and female mice aged 9-15 weeks were used for splenocyte experiments and aged 9-12 weeks were used for BMDM experiments. All experiments were approved by Monash University Animal Ethics Committee and conducted in accordance with the National Health and Medical Research Council’s, *Australian code for the care and use of animals for scientific purposes*.

### LPS-induced systemic inflammation experimental protocol

To stimulate a systemic inflammatory response, WT and IRAP KO mice were injected (i.p.) with 3 mg/kg LPS or 0.9% v/v saline as the vehicle control once for 4- or 24 hours, or once daily for 2 days (2 injections total; 48-hour endpoint). The body weight of each mouse was recorded before each injection and before culling. Whole blood was collected via cardiac puncture into heparinized tubes and centrifuged at 1,500 rpm for 10 minutes at 4°C. Plasma was stored at -80°C for later analysis. Organs including the spleen, lungs and heart were collected from each animal for further experimentation.

### Isolation of splenocytes

To isolate splenic cells, the spleen from each mouse was dissected, weighed and placed in RPMI 1640 media until being pushed through a 100 µm cell strainer in the presence of RPMI 1640. Collected cells were centrifuged at 1,500 rpm for 5 minutes at room temperature and the resulting pellet was resuspended in ammonium chloride potassium (ACK) lysis buffer (0.15 M NH_4_Cl, 0.01 M KHCO_3_, 0.0001 M EDTA-Na_2_, pH = 7.3, filtered) for 3 minutes to lyse red blood cells. Following lysis, RPMI 1640 was added to adjust the volume of each tube to 10 ml and cells were centrifuged at 1,500 rpm for 5 minutes. The white cell pellet was resuspended in 1 ml of RPMI 1640 and a cell count was conducted using a Countess automated cell counter. A 100 µl suspension containing 1 x 10^6^ splenic cells was prepared for flow cytometry. The remaining cells were frozen at -80°C in RPMI 1640 + 10% DMSO.

### Isolation and culture of BMDM

To isolate cells from the bone marrow, the tibia and femur were collected from WT and IRAP KO mice. All tissue was carefully removed from the intact bones which were then placed in complete medium consisting of RPMI-1640 supplemented with 10% heat inactivated FBS, 1% penicillin/streptomycin, 2% L-glutamine, 2% HEPES buffer and 0.1 mM 2-mercaptoethanol. The bones were transferred to a biosafety cabinet and appropriate aseptic technique was used for all following steps. The bones were soaked for 1 minute in 80% ethanol and then moved into a petri dish containing complete medium. To expose the bone marrow, the ends of each bone were removed, and bones were placed in a 0.2 ml tube with 3-4 holes at the bottom made using a 19 Ga needle. This tube was placed inside a 1.5 ml tube and subsequently centrifuged at 9,000 rpm for 2 minutes at room temperature to flush out the bone marrow. The resulting bone marrow pellet was resuspended in 200 µl of complete medium and transferred to a 15 ml tube. The suspension was incubated in 3 ml of ACK lysis buffer for 2 minutes to lyse red blood cells. Following addition of complete media to reach a total suspension volume of 10 ml, cells were centrifuged at 1,400 rpm for 4 minutes at room temperature. The pellet of white blood cells was resuspended in 10 ml of complete medium and cells were counted using a Countess automated cell counter. Cells were seeded at a density of 0.5 x 10^6^ cells/ml in 6-well plates in the presence of 10 ng/ml M-CSF in 3 ml of complete medium. For immunocytochemistry experiments, cells were grown on 13 mm coverslips in 6-well plates. Cells were maintained in an incubator at 37°C and 5% CO_2_ for 3 days. On day 3, small adherent clusters of BMDM were present. To change media, 1.5 ml of media was removed and replaced with 1.5 ml of fresh complete media with 10 ng/ml of M-CSF. On day 6 of culture, BMDM had a spindle-like morphology and were ready for treatment.

### BMDM experimental protocol

To investigate the role of IRAP in LPS-stimulated macrophage activation, BMDM from both WT and IRAP KO mice were treated for 24 hours with 100 ng/ml LPS on day 6 of culture to stimulate their differentiation into M1 macrophages. Subsets of BMDM were simultaneously treated with IRAP inhibitors (HFI-419 at 10 µM or SJM4-164 at 90 µM) at concentrations 10-fold greater than their IC_50_ for IRAP.

After 24 hours of treatment, conditioned media from each well were collected for cytokine analysis and BMDM were processed depending on the experiment. For flow cytometry and Western blot experiments, cells were gently scraped into 1.5 ml of ice-cold Dulbecco’s phosphate buffered saline (DPBS) and collected into 1.5 ml tubes. Following centrifugation at 1,400 rpm at 4°C for 4 minutes, cell pellets were either immediately stained for flow cytometry or stored at -80°C for Western blot analysis. For immunocytochemistry experiments, BMDM cultured on 13 mm coverslips were washed briefly with DPBS and then fixed for 15 minutes with 4% PFA. Fixed cells were stored at 4°C.

### Cell staining for flow cytometry

To stain cells for flow cytometry, 1 x 10^6^ splenocytes or 200 µl of BMDM resuspended in DPBS to a concentration of 0.5 x 10^6^ cells/ml, were transferred to each well of a 96-well conical bottom clear plate. The plate was centrifuged at 1,400 rpm at 4°C for 4 minutes and the resulting supernatant discarded by tipping the plate upside down. Stain mixes (see materials for antibodies used) and fluorescence minus one (FMO) controls were prepared in flow cytometry buffer (DPBS + 2% heat inactivated FBS) and 30 µl was added to the appropriate well. Cells were resuspended well and incubated in the dark for 15 minutes. Cells designated as unstained controls were resuspended in flow cytometry buffer alone. After the 15-minute incubation, 150 µl of flow cytometry buffer was added to each well to flood the stain and the plate was centrifuged at 1,400 rpm at 4°C for 4 minutes. The resulting supernatant was discarded by tipping the plate upside down. Cells were resuspended in 30 µl of viability stain (zombie dye; 1:500 concentration) except for the unstained and FMO controls and incubated for 15 minutes in the dark. Flow cytometry buffer (150 µl) was added to each well and the plate was centrifuged again at 1,400 rpm at 4°C for 4 minutes. The cell pellet was fixed by resuspension in 60 µl of 1% PFA (diluted from 4% PFA with DPBS). Stained cells were transferred into separate labeled flow tubes and stored at 4°C until flow cytometry was conducted.

### Acquisition and analysis of flow cytometry data

All flow cytometry data was acquired using Fortessa X20 flow cytometers (BD Biosciences) at Flowcore (Monash University) and analyzed using FlowJo (Tree Star, Ashland, OR). The gating strategy described in **Supplementary Figure 4** was used in all splenocyte flow cytometry experiments. Firstly, all cells were gated based on their size and granularity using FSC-A/SSC-A. Single cells were then gated using FSC-A/FSC-H. Live cells were then gated using Zombie/SSC-A where live cells were negative for zombie/viability staining. Immune cell populations including CD45^+^ CD11b^+^ myeloid cells, CD11c^+^ DCs, CD45^+^ F4/80^+^ CD206^-^ M1 macrophages, CD45^+^ F4/80^+^ CD206^+^ M2 macrophages, CD4^+^ CD45^+^ T helper cells and CD19^+^ CD45^+^ B cells, were subsequently gated. The frequencies of each cell population were analyzed as well as the geometric means of the activation markers CD40, CD80, CD86 and MHCII in all myeloid cell populations.

The gating strategy described in **Supplementary Figure 4** was used in all BMDM flow cytometry experiments. Firstly, all cells were gated based on their size and granularity using FSC-A/SSC-A. Single cells were then gated using FSC-A/FSC-H. Live cells were then gated using Zombie/SSC-A where live cells were negative for zombie/viability staining. Immune cell populations including CD45^+^ F4/80^+^ CD206^-^ M1 macrophages, CD11c^+^ DCs and CD11c^-^ MHCII^+^ Gr-1^+^ MDSCs, were subsequently gated. It is worth noting that myeloid progenitor cells isolated from the bone marrow and cultured in the presence of M-CSF are expected to consist predominantly (>90%) of CD11b^+^ F4/80^+^ macrophages (15). Therefore, there were very few other immune cell populations examined in this study. The frequencies of each cell population were analyzed as well as the geometric means of the activation markers CD40, CD80, CD86 and MHCII in macrophage populations.

### Cytokine ELISAs

The expression of the pro-inflammatory cytokines TNF-α, IL-1β and IL-6 in the plasma of vehicle- or LPS-treated mice or secreted by BMDM in the conditioned media was measured using mouse DuoSet ELISA kits (R&D Systems). ELISAs were run according to the manufacturer’s instructions.

### Immunocytochemistry

To visualize the expression of various cytokines in CD45^+^ BMDM, immunocytochemistry was conducted. Cells fixed with 4% PFA were washed 3 times for 10 minutes each with 1x PBS. Coverslips were removed from the 6-well plates and placed on a microscope slide, with the cells facing upwards. Cells were blocked and permeabilized with 10% normal goat serum/0.2% triton X-100 for 1 hour, followed by overnight incubation at room temperature with the anti-CD45, anti-TNFα, anti-IL-1β, anti-IL-6, anti-IRAP or anti-TLR4 antibody diluted in antibody diluent (all 1:500 dilution). For dual labeling, the cells were incubated with both primary antibodies overnight. The following day, the primary antibody was removed and the cells were rinsed with PBS in a series of three washes (10 minutes each). The cells were subsequently incubated with the appropriate secondary antibody for 2 hours at room temperature (1:500 dilution) and the three washes with PBS were repeated. One drop of Vectashield mounting medium containing DAPI was placed onto each coverslip and a rectangular coverslip was secured on top. After ensuring no bubbles were present, the mounting medium was left to dry for 10 minutes before imaging.

### Imaging and analysis

All imaging was performed using a Zeiss Axio Imager M1. At least 6-8 images at 20x magnification were taken per treatment group, with each field of view containing cells at approximately the same level of confluency. An ImageJ macro was developed to quantify the positive immunofluorescent staining area of TLR4 in a field of view. This number was normalized to the total cell count for the same image, averaged for each group and presented relative to the mean of the male WT control group. The cell counts, which used thresholded-DAPI stained nuclei, were completely automated as part of the ImageJ macro.

To quantify the colocalization of two proteins such as IRAP and TLR4, an ImageJ macro was written to measure the intersection using the “AND” option in the image calculator tool in a given field of view. This number was then normalized to the total positive staining area in the same image, calculated as the combined area measured using the “Add” option in the image calculator tool minus the intersection, to give percent area of colocalization. The percent area colocalization was finally averaged for each group.

### Western blotting

To measure the protein expression of IRAP in BMDM from WT and IRAP KO mice, standard Western blotting was conducted. To extract the protein, BMDM cell pellets stored at -80°C were thawed and lysed in 40 µl of buffer consisting of 0.5% triton X-100 and 1% protease/phosphatase inhibitor cocktail in 50 mM Tris/150 mM NaCl. Following a 30-minute incubation on ice, samples were centrifuged (6,000 rpm for 10 minutes at 4°C) and the protein-containing supernatants were transferred to new tubes.

All experiments were conducted under non-reducing conditions where lysates were diluted in 1.5x laemmli buffer (without 2-mercaptoethanol) and heated at 70°C for 5 minutes. For BMDM lysates, ~80-100 µg of protein was loaded per well (calculated retrospectively by measurement of the protein concentration of diluted lysates using a nanodrop). Before loading of samples, 10% gels (15 wells, 1mm thickness) were prepared using a TGX Stain-free FastCast Acrylamide starter kit and standard Western blotting protocol was followed whereby protein separated in the gel is transferred to a membrane, blocked with 5% skim milk for 1 hour, incubated with anti-IRAP antibody overnight at 4°C and the appropriate HRP-conjugated secondary antibody the following day. Individual protein bands were quantified by measuring the optical density (OD) per unit area using ImageLab software. Correction for differences in protein per well was normalized to the protein concentration measured using a nanodrop. All protein expression is presented as a relative ratio to the mean of the male WT control group to allow for examination of fold changes.

### Statistical analysis

Results are presented as mean ± SEM and statistical analysis was conducted using GraphPad Prism software. In the *in vivo* LPS model, two-way ANOVAs with Tukey’s multiple comparisons were used to investigate the effect of LPS or genotype on the body weights of mice, the frequency of different cell populations and the expression of different activation markers. The main effects, namely treatment (vehicle vs LPS) and genotype (WT vs KO) as well as the interaction between the two factors, generated from the two-way ANOVAs, are presented in **Supplementary Table 1** whereas results from the *post-hoc* Tukey’s multiple comparison are presented in the figures in the main results text. It is worth noting there is some variability in the basal frequencies of some of the immune cell populations at different timepoints, potentially due to them being conducted on different days. To compare the spleen weights of all groups, a three-way ANOVA with Tukey’s *post-hoc* test was used with LPS treatment, genotype and sex as the independent variables. To determine if there was a relationship between the change in body weight and spleen weight, correlational analysis with Pearson’s correlation coefficient was conducted using a two-way ANOVA with Tukey’s *post-hoc* analysis. The quantification of cytokine expression in the plasma of vehicle- and LPS-treated mice, measured using sandwich ELISAs, was analyzed by two-way ANOVAs with Tukey’s *post-hoc* test with LPS treatment and genotype as the independent variables.

In the *in vitro* BMDM model, the frequency of different cell populations and the expression of activation markers across groups were analyzed using a two-way ANOVA with Tukey’s *post-hoc* analysis with the LPS treatment and genotype as independent variables. The quantification of cytokine expression in the conditioned media of BMDM, measured using sandwich ELISAs, was analyzed by three-way ANOVAs with Tukey’s *post-hoc* test with LPS treatment, genotype and sex as the independent variables. In Western blot experiments, a three-way ANOVA with Tukey’s *post-hoc* test was used to compare IRAP protein expression across groups in BMDM with independent variables of LPS treatment, genotype and sex. A two-way ANOVA with Tukey’s *post-hoc* was used to compare IRAP and CD45 colocalization in immunocytochemistry experiments. To compare the expression of TLR4 in BMDM, colocalization of IRAP or TLR4 in BMDM and expression of TLR4 at the cell surface in M1 macrophages in the spleen, three-way ANOVAs with Sidaks’s *post-hoc* analysis were conducted.

## Results

### LPS treatment results in time-dependent losses in body weight

**Figure 1:**
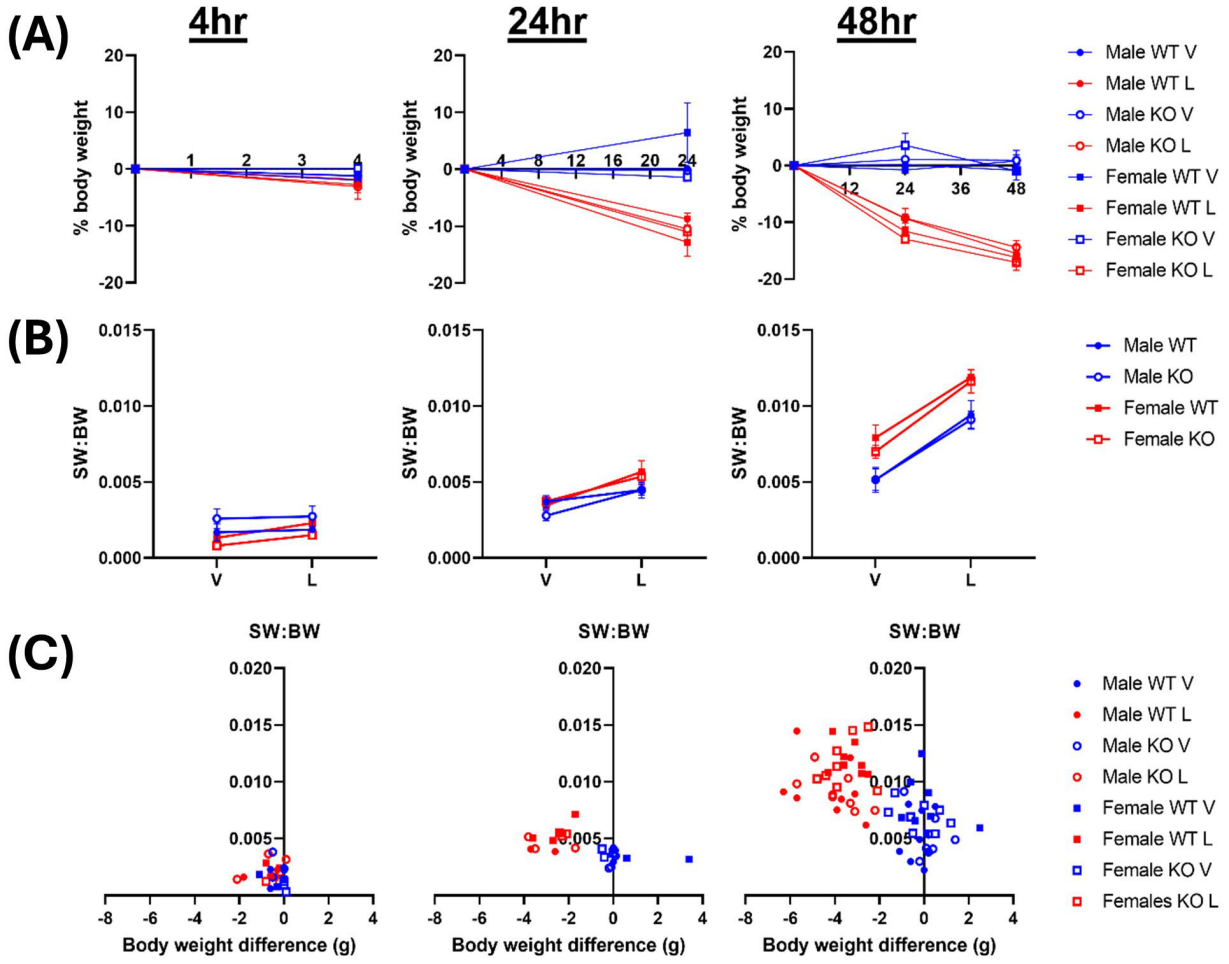
LPS treatment results in time-dependent losses in body weight. Male & female wildtype (WT; filled circle) & IRAP knockout (KO; empty circle) mice (aged 10-15 weeks) were administered either vehicle (V; blue) or LPS (L; red) once for 4 hours (n=3) or 24 hours (n=3) or twice over 48 hours (n=8). **(A)** Body weight (BW), presented as % change from before treatment, decreased following LPS administration. **(B)** Spleen weight relative to the body weight (SW: BW) increased with LPS treatment. **(C)** Correlation analysis between change in BW and SW: BW after LPS treatment for 4 hours (r = 0.38, R^2^ = 0.14, p = 0.23), 24 hours (r = -0.62, R^2^ = 0.39, p = 0.03) and 48 hours (r = -0.74, R^2^ = 0.55, p<0.0001), analyzed via Pearson correlation. Data is presented as the mean ± SEM.

There were no notable genotype or sex differences in body weight, spleen weight or clinical severity scores after a single intraperitoneal dose of LPS for either 4- or 24-hours, or two daily doses over 48-hours. LPS treatment induced a significant reduction in body weight across both genotypes at each timepoint, with a mean loss of 2.4 ± 0.3% at 4 hours (p = 0.0126), 10.8 ± 0.8% at 24 hours (p < 0.0001) and 15.8 ± 0.6% at 48 hours (p < 0.0001, vs baseline, two-way ANOVAs; **Figure 1A**). LPS-treated mice all displayed a greater clinical severity score, with decreased locomotor activity and lack of response to handling, similar to previous reports in this sepsis model (13, 16). Significant time-dependent, progressive increases in the spleen weight relative to the body weight were recorded in all LPS-treated groups compared to vehicle controls after 24-hours (p = 0.1360 (4hr), p < 0.0001 (24hr), p < 0.0001 (48hr), three-way ANOVAs; **Figure 1B**), regardless of genotype. There was also a significant inverse correlation between the body weight change and spleen weight 24 hours after LPS treatment, with mice that lost more body weight having larger spleens (R^2^ = 0.14, p = 0.23 (4hr), R^2^ = 0.39, p = 0.03 (24hr), R^2^ = 0.55, p<0.0001 (48hr); **Figure 1C**). Together, these findings validate the systemic inflammation model, confirming that LPS treatment causes significant weight loss as well as inducing an increase in immune cell infiltration into the spleen.

### IRAP influences the activation of dendritic cells in the spleen of female mice in response to LPS

DCs participate in all stages of gram-negative sepsis initiation and progression, with documented alterations in their frequency, differentiation and activation state (17). IRAP is highly expressed in DCs where it has been shown to play a number of physiological roles (7, 11, 18). The effect of global gene deletion of IRAP on the acute inflammatory response to LPS treatment was investigated by comparing changes in splenic DC frequency and activation, via the markers CD40, CD80, CD86 and MHCII, in WT and IRAP KO, male and female mice. In control male mice, 2.9 ± 0.6% of cells in the spleen were CD11c^+^ DCs, while in control female mice, 3.4 ± 0.9% of cells were DCs. These frequencies are similar to those reported in previous studies in C57BL/6 mice (19, 20). **Supplementary Table 1** summarizes the analysis from the two-way ANOVA showing either an overall treatment (vehicle vs LPS) or genotype (WT vs IRAP KO) effect on the distinct immune cell frequencies or the activation markers. A significant LPS treatment effect on the frequency of the DCs was detected in both sexes at 24 hours (**Supplementary Table 1**). *Post-hoc* analysis revealed that this significant decrease in DC frequency was only observed in the WT group in the female mice (p = 0.0039, two-way ANOVA Tukey’s *post-hoc* test; **Supplementary Figure 1B**). Furthermore, in female mice, there were significantly greater fold-increases in CD40, CD86 and MHCII markers in DCs following LPS stimulation at 24 hours (**Supplementary Table 1**; **Supplementary Figure 1C**) and by 48h, the most of these activation markers were normalized to levels closer to or even lower than that of vehicle controls. Overall, the only significant genotype effect in DCs were on the activation markers in cells isolated from spleens of female mice 24 hours after LPS stimulation (**Supplementary Table 1**). This suggests a sex-dependent role for IRAP in moderating the DC response to LPS induced inflammation.

### IRAP gene deletion alters the responsiveness of macrophages to acute LPS stimulation in male mice

Macrophages have diverse roles in all phases of gram-negative sepsis as part of both the innate and adaptive immune response (21). Unlike other immune cell types, they also have the unique ability to become polarized into a pro-inflammatory M1 phenotype or anti-inflammatory M2 phenotype depending on cues in the microenvironment (21). LPS is a potent stimulator of M1 macrophage activation *in vitro* (22). While the categorization of M1 and M2 macrophages is important experimentally to gain insights into their functional roles, the expressions of many macrophage markers are continually changing suggesting that macrophage polarization is a dynamic process.

**Figure 2:**
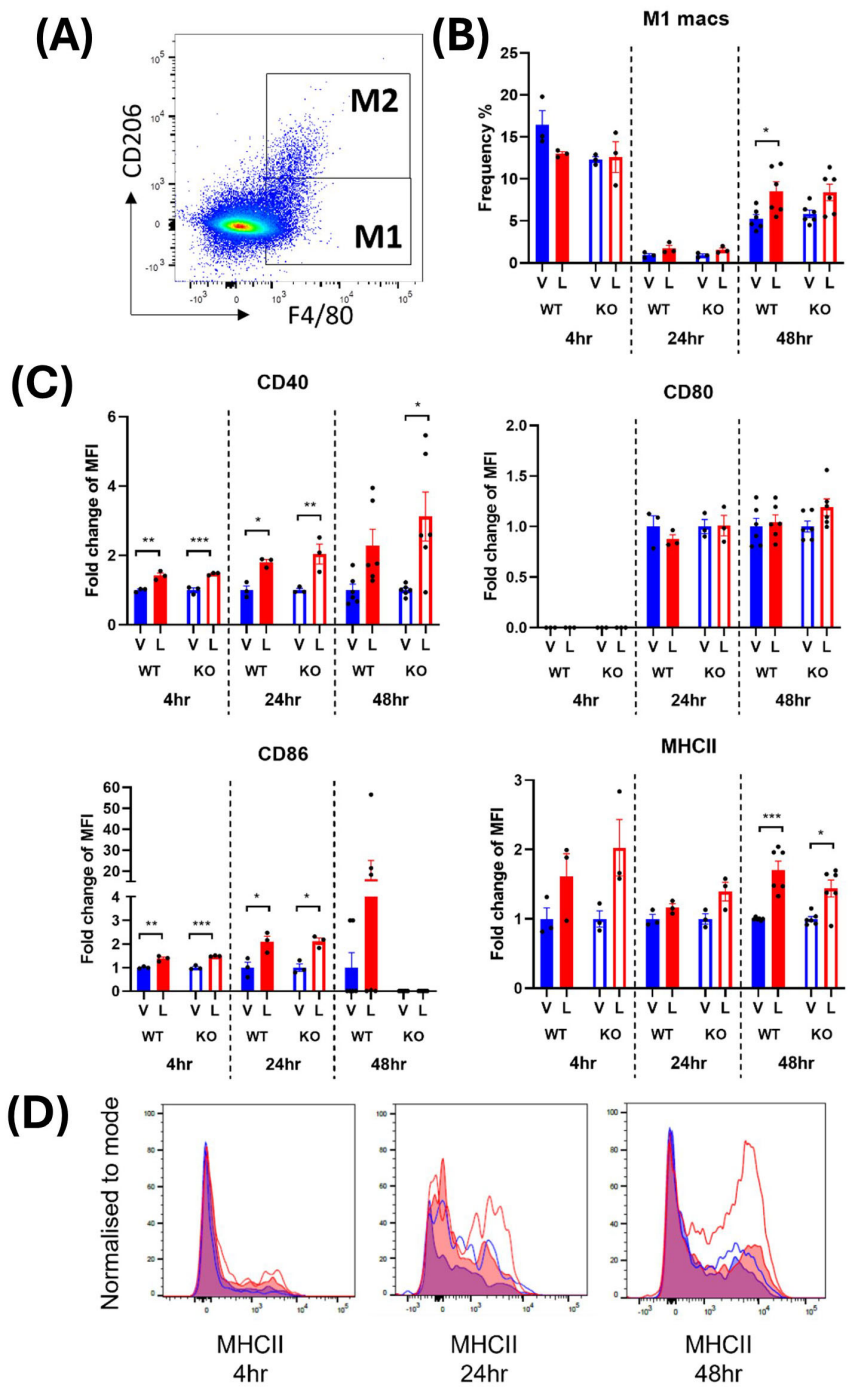
IRAP gene deletion may result in heightened responsiveness of M1 macrophages in the spleen of male mice 48 hours following LPS treatment. **(A)** Representative flow cytometry dot plot showing the gating for CD45^+^ F4/80^+^ CD206^-^ M1 macrophages in the spleen, 48-hours following vehicle treatment. **(B)** The frequency (%) among live CD45^+^ cells of M1 macrophages in the spleen of male wildtype (WT; filled bars) and IRAP knockout (KO; empty bars) mice (aged 10-15 weeks) administered either vehicle (V; blue) or LPS (L; red) once for 4 hours (n=3) or 24 hours (n=3) or twice over 48 hours (n=6). **(C)** Quantification of the fold changes in mean fluorescence intensity (MFI) compared to the mean of vehicle controls of the activation markers CD40, CD80, CD86 and MHCII in M1 macrophages. Note that no positive MFI values were measured for CD80 at 4 hours. **(D)** Frequency curves of MHCII in macrophages from spleens of vehicle- (blue) and LPS-treated (red) WT (filled curves) and IRAP KO (empty curves) mice. Data from each timepoint was analyzed separately using a two-way ANOVA with Tukey’s post-hoc test, *p<0.05, **p<0.01, ***p<0.001. All data is presented as mean ± SEM.

**Figure 3:**
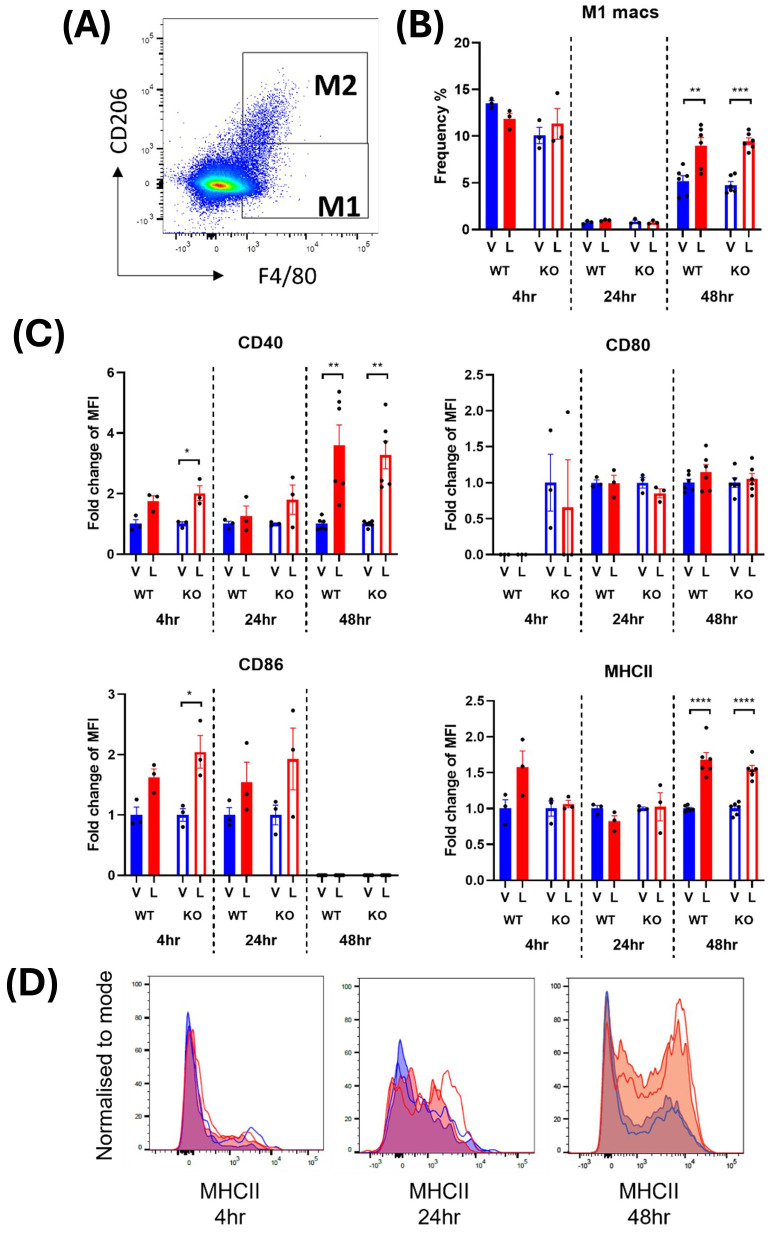
IRAP gene deletion does not appear to influence the LPS-responsiveness of M1 macrophages in the spleen of female mice. **(A)** Representative flow cytometry dot plot showing the gating for CD45^+^ F4/80^+^ CD206^-^ M1 macrophages in the spleen, 48-hours following vehicle treatment. **(B)** The frequency (%) among live CD45^+^ cells of M1 macrophages in the spleen of female wildtype (WT; filled bars) and IRAP knockout (KO; empty bars) mice (aged 10-15 weeks) administered either vehicle (V; blue) or LPS (L; red) once for 4 hours (n=3) or 24 hours (n=3) or twice over 48 hours (n=6). **(C)** Quantification of the fold changes in mean fluorescence intensity (MFI) compared to the mean of vehicle controls of the activation markers CD40, CD80, CD86 and MHCII in M1 macrophages. **(D)** Frequency curves of MHCII in macrophages from spleens of vehicle- (blue) and LPS-treated (red) WT (filled curves) and IRAP KO (empty curves) mice. Data from each timepoint was analyzed separately using a two-way ANOVA with Tukey’s post-hoc test, *p<0.05, **p<0.01, ***p<0.001, ****p<0.001. All data is presented as mean ± SEM.

M1 macrophages were defined as CD45^+^ monocytes that expressed F4/80 and lacked the M2 macrophage marker, CD206 (**Figures 2A**, **3A**). In male mice, LPS treatment significantly increased the frequency of M1 macrophages in the spleen at 24 and 48 hours from a mean across genotypes of 0.91 ± 0.007% to 1.64 ± 0.09% and 5.55 ± 0.29% to 8.47 ± 0.06%, respectively (**Supplementary Table 1**; **Figure 2B**). In females, the increase in M1 frequency was only significant at 48 hours post LPS treatment from 4.98 ± 0.21% to 9.20 ± 0.24% (**Supplementary Table 1**; **Figure 3B**). No significant genotype effect was detected in the frequency of M1 macrophages or expression of activation markers CD40, CD80, CD86 or MHCII in either sex (**Supplementary Table 1**). Further examination of the activation markers using *post-hoc* analysis revealed a marginal increase in CD40 expression in M1 macrophages from the male IRAP KO mice (p = 0.0124, two-way ANOVA Tukey’s *post-hoc* test) (**Figure 2C**). Interestingly, evaluation of the frequency curves revealed that in male IRAP KO mice, there appears to be a higher frequency of M1 macrophages expressing MHCII at 24 hours and more so at 48 hours after LPS treatment (**Figure 2D**), an effect that was not present in female mice (**Figures 3A, C, D**).

**Figure 4:**
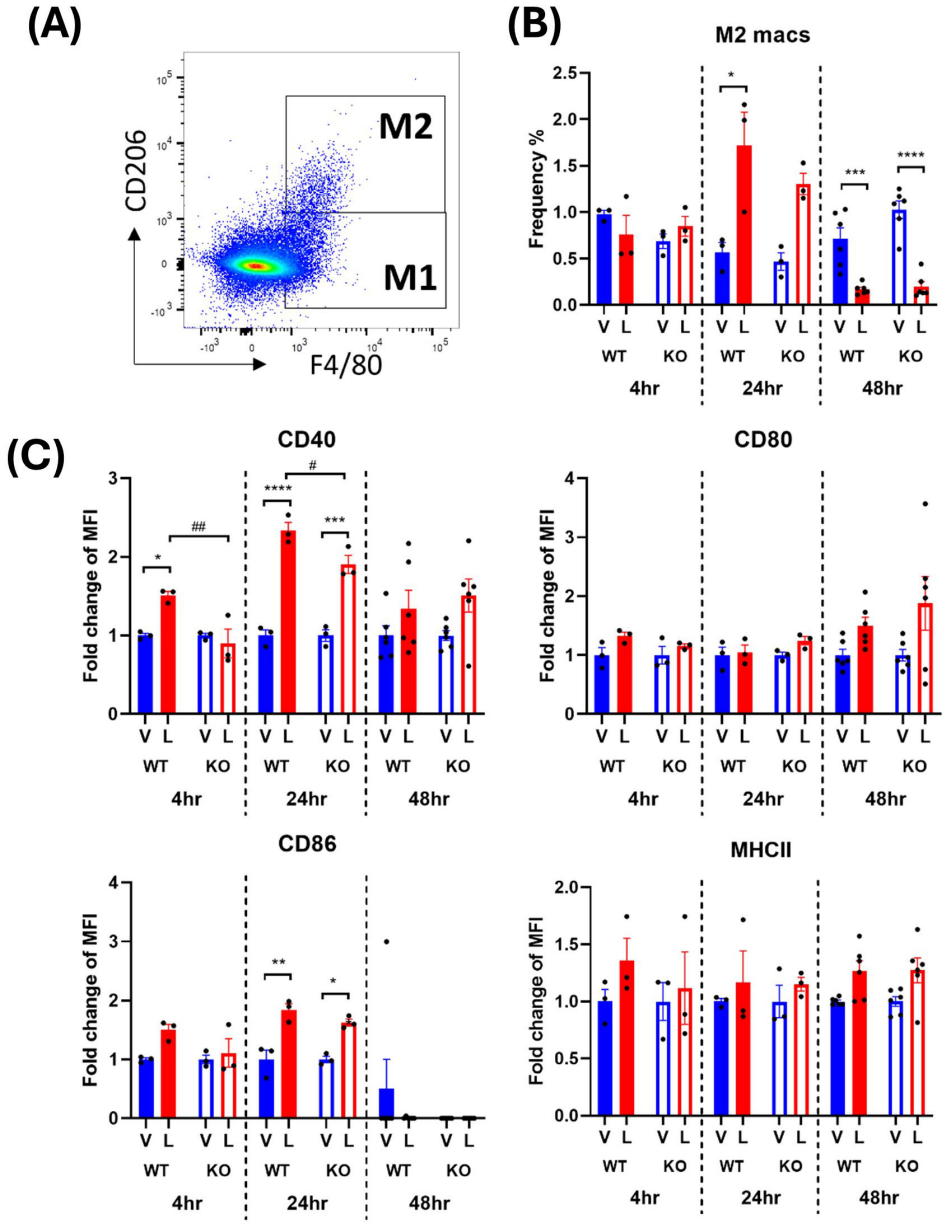
IRAP gene deletion may influence the LPS responsiveness of M2 macrophages in the spleen of male mice. **(A)** Representative flow cytometry dot plot showing the gating for CD45^+^ F4/80^+^ CD206^+^ M2 macrophages in the spleen, 48-hours following vehicle treatment. **(B)** The frequency (%) among live CD45^+^ cells of M2 macrophages in the spleen of male wildtype (WT; filled bars) and IRAP knockout (KO; empty bars) mice (aged 10-15 weeks) administered either vehicle (V; blue) or LPS (L; red) once for 4 hours (n=3) or 24 hours (n=3) or twice over 48 hours (n=6). **(C)** Quantification of the fold changes in mean fluorescence intensity (MFI) compared to the mean of vehicle controls of the activation markers CD40, CD80, CD86 and MHCII in M2 macrophages. Data from each timepoint was analyzed separately using a two-way ANOVA with Tukey’s post-hoc test, *p<0.05, **p<0.01, ***p<0.001, ****p<0.0001 vehicle vs LPS, #p<0.05, ##p<0.01 WT vs KO. All data is presented as mean ± SEM.

**Figure 5:**
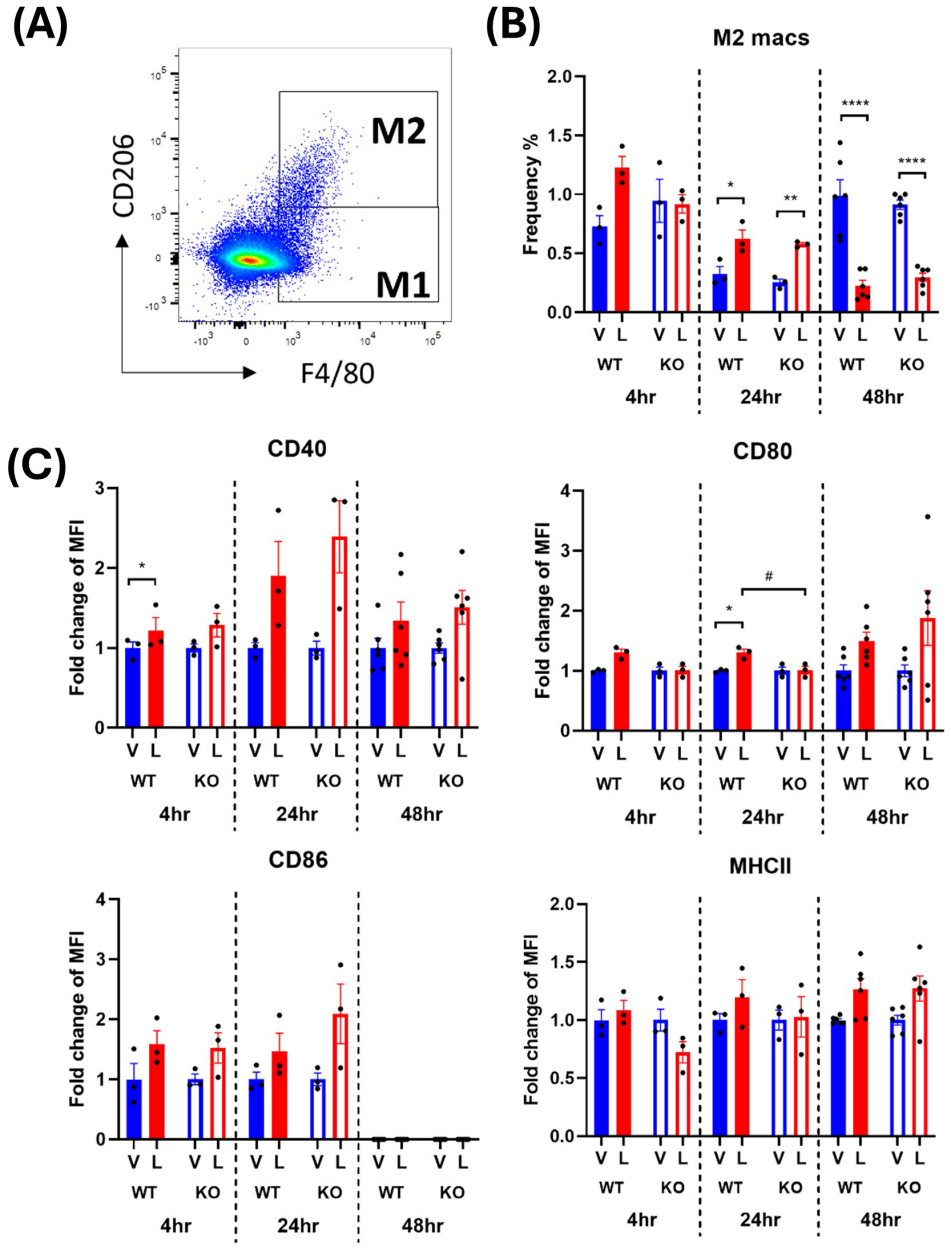
IRAP gene deletion does not appear to influence the LPS responsiveness of M2 macrophages in the spleen of female mice. **(A)** Representative flow cytometry dot plot showing the gating for CD45^+^ F4/80^+^ CD206^+^ M2 macrophages in the spleen, 48-hours following vehicle treatment. **(B)** The frequency (%) among live CD45^+^ cells of M2 macrophages in the spleen of female wildtype (WT; filled bars) and IRAP knockout (KO; empty bars) mice (aged 10-15 weeks) administered either vehicle (V; blue) or LPS (L; red) once for 4 hours (n=3) or 24 hours (n=3) or twice over 48 hours (n=6). **(C)** Quantification of the fold changes in mean fluorescence intensity (MFI) compared to the mean of vehicle controls of the activation markers CD40, CD80, CD86 and MHCII in M2 macrophages. Data from each timepoint was analyzed separately using a two-way ANOVA with Tukey’s post-hoc test, *p<0.05, **p<0.01, ****p<0.0001 vehicle vs LPS, #p<0.05 WT vs KO. All data is presented as mean ± SEM.

Activated M2 macrophages were defined as CD45^+^ monocytes expressing F4/80 and CD206 (**Figures 4A**, **5A**). The frequency of the M2 macrophages and their expression of activation markers after an LPS challenge were evaluated. The earliest significant changes in M2 macrophages were detected at the 24-hour timepoint where a transient 2-3-fold increase in cell frequency in response to LPS was followed by 4-5-fold decrease at 48 hours (**Figures 4B**, **5B**). This suggests that the environment encountered by infiltrating monocytes in the spleen promoted polarization to an M2 anti-inflammatory phenotype at 24 hours which transformed to the M1 pro-inflammatory phenotype at 48 hours. This trend was consistent across males and females (**Figures 4B**, **5B**). However, in male WT mice, an exaggerated increase in M2 frequency in response to LPS was detected at 24 hours compared to the IRAP KO. This pattern was completely reversed at 48 hours after LPS treatment where the frequency was significantly decreased in both genotypes and sexes (**Figures 4B**, **5B**). As for the expression of the activation markers, IRAP gene deletion significantly dampened the LPS-induced increases in CD40 at the 24-hour timepoint but only in male mice (**Figures 4C**, **5C**).

### IRAP does not influence the frequency of helper T cells or B cells in response to LPS in male mice

Gram-negative sepsis results in dysregulation and deficits in the adaptive immune response, with depletion of functional T and B cells due to increased cell death or exhaustion (23). IRAP is known to participate in specific components of the adaptive immune response in T cells (10), although there has been no investigation into IRAP in B cells. In the spleens of vehicle-treated mice across both genotypes, 8.79 ± 1.02% of cells were CD4^+^ CD45^+^ helper T cells in males (**Supplementary Figure 2B**) and 11.21 ± 1.34% of cells were helper T cells in females (**Supplementary Figure 2C**). There was also a very high proportion of CD19^+^ CD45^+^ B cells in the spleens of control mice across genotypes, with a frequency of 35.26 ± 2.11% in males and 37.21 ± 2.50% in females, similar to previous reports (20). There were significant decreases in helper T cell frequency 24 and 48 hours following LPS treatment in both males and females (**Supplementary Table 1**). This supports reports on T cell depletion in sepsis (24). Notably, there was also a significant genotype effect in T cell frequency at all timepoints in female mice (**Supplementary Table 1**, **Supplementary Figure 2C**). LPS administration induced significant increases in B cell frequency in both sexes at 48 hours (**Supplementary Table 1**) but no genotype effect was observed.

### Pro-inflammatory cytokines increase following LPS administration

The altered secretion of cytokines from cells such as macrophages is a key characteristic of gram-negative sepsis with an uncontrolled overproduction of pro-inflammatory mediators in acute phases (3). There were overall increases in pro-inflammatory tumor necrosis factor (TNF)-α, interleukin (IL)-1β and IL-6 in the plasma of LPS-treated compared with vehicle-treated mice at the 4-hour timepoint (**Supplementary Figure 3**), although only IL-1β and IL-6 were statistically significant in both males (IL-1β: p=0.0224, IL-6: p=0.0191; two-way ANOVAs) and females (IL-1β: p=0.0020, IL-6: p=0.0058; two-way ANOVAs). There were no significant genotype effects in either sex. It is worth noting that while essentially none of the cytokines were detected in any plasma samples from control mice, LPS induced 10-to-100-fold increases in protein levels of TNFα and IL-1β and 1000-fold increases in protein levels of IL-6. Pro-inflammatory cytokines levels were subsequently restored to undetectable control levels at the 24- and 48-hour timepoints following LPS stimulation. This observation is supported by a previous study where cytokine production occurred within 2 hours of LPS injection with peak increases in circulating TNF-α and IL-6 detected at this time point before returning to baseline over the following 6 hours (25).

### IRAP gene deletion results in a heightened pro-inflammatory response in male BMDM

Given the complexity of the acute inflammatory response *in vivo* and the plethora of factors that could contribute to the regulation of macrophage activation, it is difficult to ascertain whether locally produced IRAP within the macrophages themselves plays a role in LPS-responsiveness and activation state. To explore this concept under controlled conditions *in vitro*, bone-marrow derived macrophages (BMDM) were treated with LPS to stimulate polarization into an M1 pro-inflammatory phenotype (26, 27).

**Figure 6:**
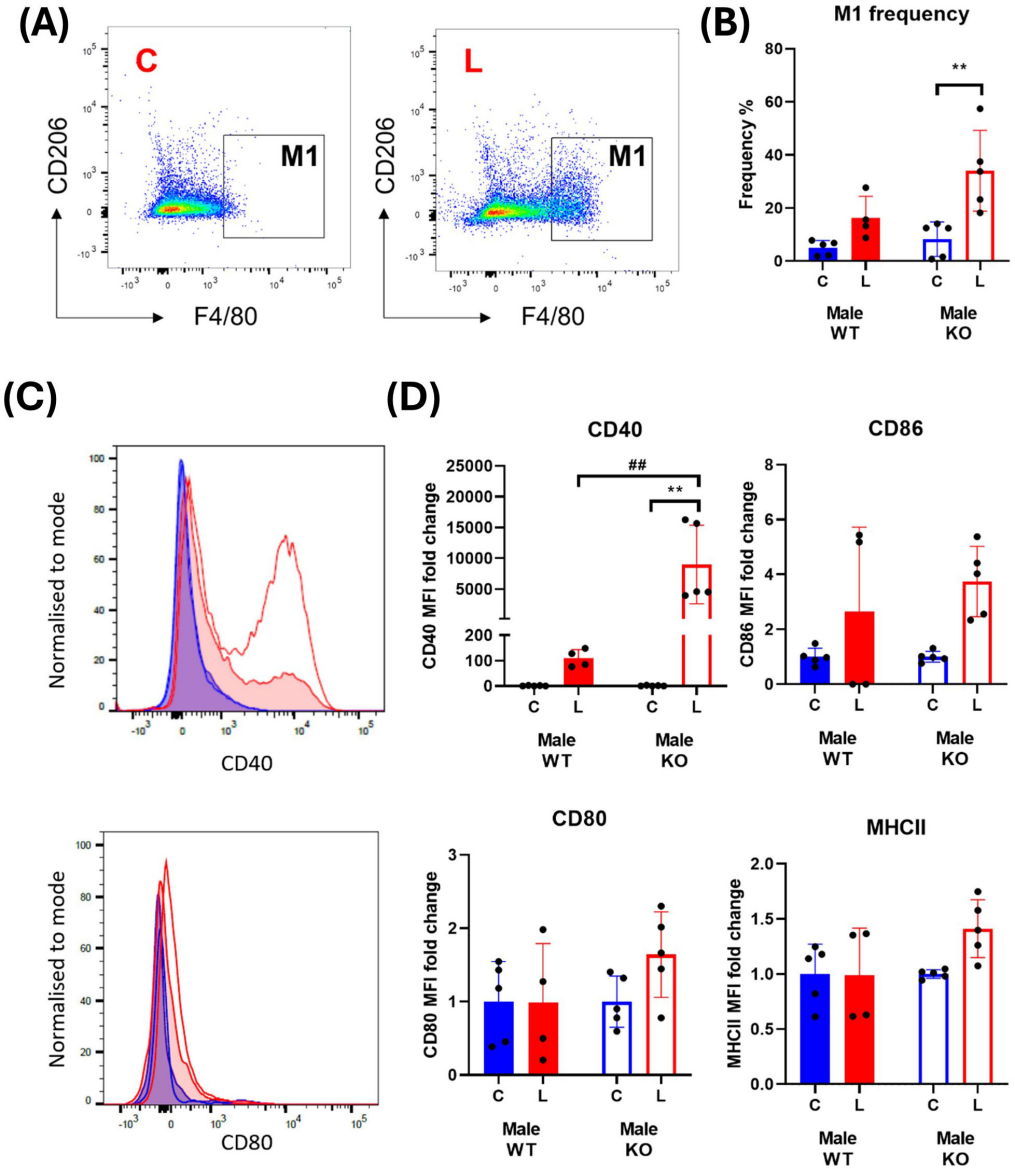
IRAP gene deletion results in a heightened pro-inflammatory response in male BMDM. **(A)** Representative flow cytometry dot plots showing control **(C)** and LPS-treated (L) CD45^+^ F4/80^+^ CD206^-^ M1 macrophages from male wildtype (WT) mice and **(B)** the frequencies (%) of the M1 macrophage population in WT (filled bars) and IRAP knockout (KO; empty bars) BMDM cultures. **(C)** Frequency curves of the activation markers CD40 and CD80 in C (blue) and L (red) WT (filled curve) and IRAP KO (empty curve) M1 macrophages. **(D)** Quantification of the fold changes in mean fluorescence intensity (MFI) compared to the mean of the controls of CD40, CD80, CD86 and MHCII in M1 macrophages. Data was analyzed using a two-way ANOVA with Tukey’s post-hoc test, **p<0.01 control vs LPS, ##p<0.01 WT vs KO, n=4-5. All data is presented as mean ± SEM.

Examination of BMDM from male WT mice revealed that LPS stimulation increases the proportion of M1 macrophages from 4.98 ± 1.24% to 16.34 ± 4.04% (p = 0.3019, two-way ANOVA; **Figure 6A, B**). Interestingly, this response was exacerbated in cells isolated from male IRAP KO mice with an increase from 8.27 ± 2.91% to 34.03 ± 6.80% (p=0.0029, two-way ANOVA; **Figure 6B**). This heightened response in cells deficient for IRAP was mirrored in the expression of the activation marker, CD40, following LPS treatment with a significant genotype (p = 0.0102) and treatment effect (p = 0.0089) as well as a significant interaction between the two factors (p=0.0102, two-way ANOVA; **Figure 6D**). There were inconsistent, non-significant LPS-mediated effects on the expression of CD80, CD86 and MHCII (**Figures 6C, D**). Notably, treatment with small molecule IRAP inhibitors developed by our laboratory, HFI-419 (28) and SJM4-164 (unpublished), did not exert any consistent effects on any of the measured variables (data not shown), supporting previous findings of the lack of the catalytic site involvement in regulating the immune response (7, 10). Overall, IRAP deficiency resulted in an increased frequency of M1 macrophages and expression of CD40 suggesting that the enzyme plays a role in dampening M1 macrophage activation thus controlling the pro-inflammatory response to an acute immune challenge.

**Figure 7:**
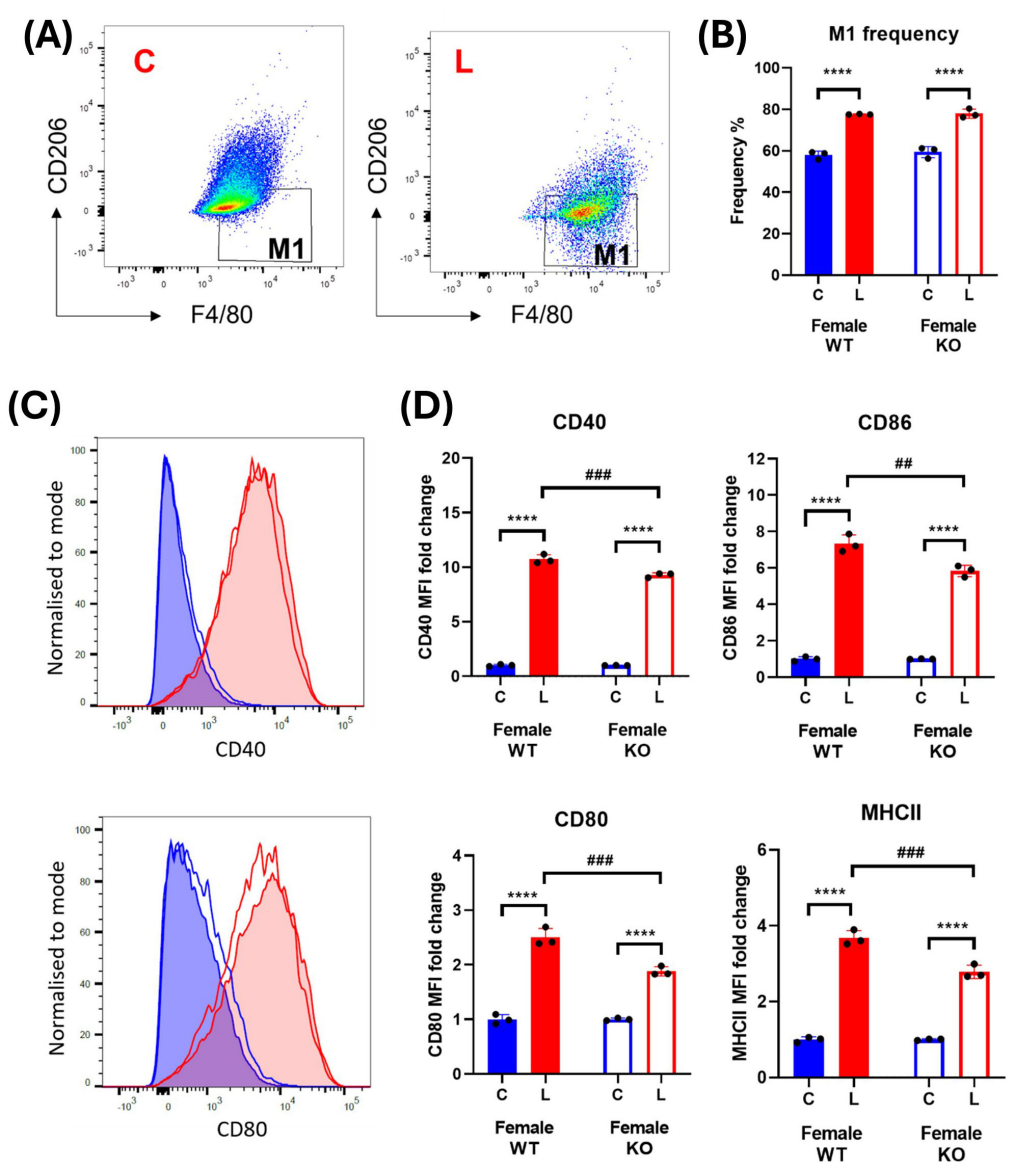
IRAP gene deletion does not significantly alter macrophage activation in female BMDM. **(A)** Representative flow cytometry dot plots showing control **(C)** and LPS-treated (L) CD45^+^ F4/80^+^ CD206^-^ M1 macrophages from female wildtype (WT) mice and **(B)** the frequencies (%) of the M1 macrophage population in WT (filled bars) and IRAP knockout (KO; empty bars) BMDM cultures. **(C)** Frequency curves of the activation markers CD40 and CD80 in C (blue) and L (red) WT (filled curve) and IRAP KO (empty curve) M1 macrophages. **(D)** Quantification of the fold changes in mean fluorescence intensity (MFI) compared to the mean of the controls of CD40, CD80, CD86 and MHCII in M1 macrophages. Data was analyzed using a two-way ANOVA with Tukey’s post-hoc test, ****p<0.0001 control vs LPS, ##p<0.01, ###p<0.001 WT vs KO, n=3. All data is presented as mean ± SEM.

Unlike the macrophages derived from male mice, LPS-induced significant and consistent increases in the frequency of M1 macrophages, as well as in all the activation markers, of female mice (**Figure 7**). However, IRAP gene deletion resulted in a significant suppression of the LPS response on these markers in the BMDM (**Figure 7C**).

**Figure 8:**
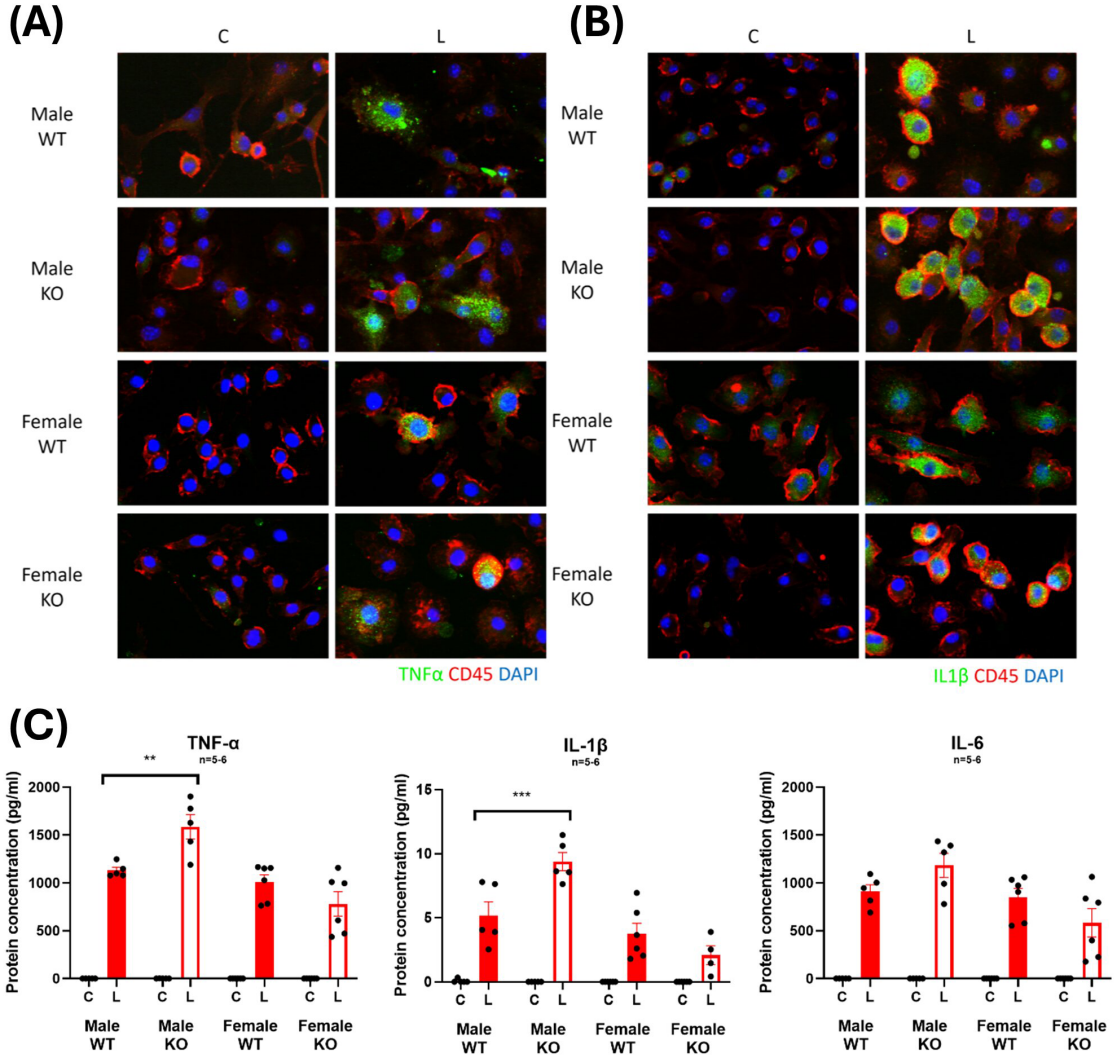
IRAP-deficient BMDM have heightened LPS-induced increases in TNF-α and IL-1β expression. Representative immunofluorescent staining of **(A)** TNF-α or **(B)** IL-1β (green) in control **(C)** or LPS-treated (L) CD45^+^ (red) BMDM from male and female wildtype (WT) and IRAP knockout (KO) mice. **(C)** Protein concentrations (pg/ml) of TNF-α, IL-1β and IL-6 in the conditioned media of BMDM measured using a sandwich ELISA. Data was analyzed using three-way ANOVAs with Tukey’s post-hoc test, **p<0.01, ***p<0.001, n=5-6. Data is presented as mean ± SEM.

Furthermore, sandwich ELISAs conducted on conditioned media from BMDMs revealed significant increases in the secretion of the pro-inflammatory cytokines, TNF-α and IL-1β with LPS treatment from cells derived from male WT and IRAP KO mice (**Figure 8**) with a heightened TNF-α and IL-1β secreted in the IRAP deficient BMDM. This heightened response was not observed in cells derived from female mice. These results provide a clearer picture of the role of IRAP on the production and secretion of pro-inflammatory cytokines from macrophages in response to LPS stimulation.

### IRAP expression and distribution changes with LPS stimulation in BMDM

**Figure 9:**
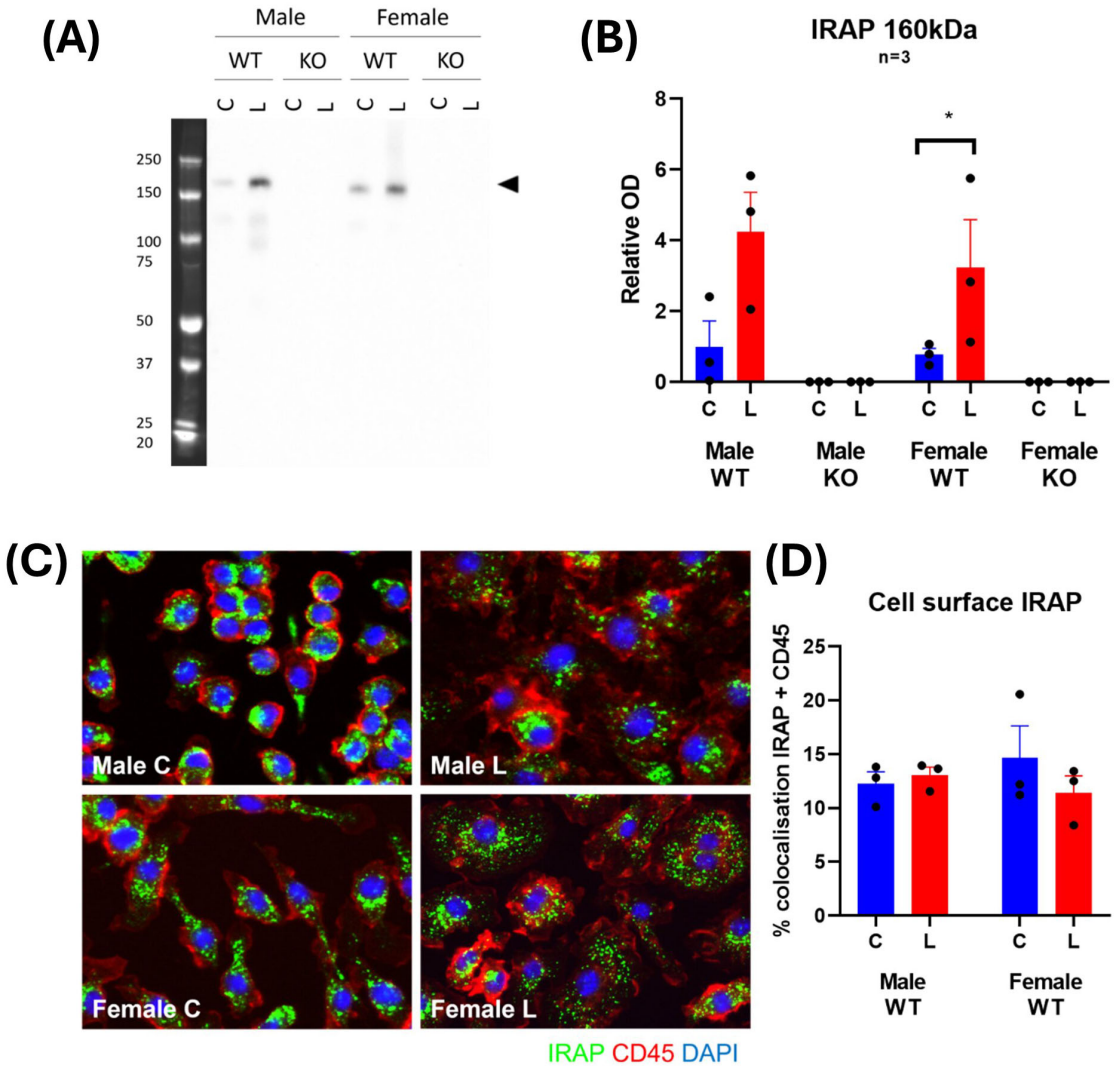
IRAP expression and distribution changes with LPS stimulation in BMDM. **(A)** Representative Western blot and **(B)** densitometric quantification of IRAP (~160 kDa) in cell lysates of untreated control (C; blue) or LPS-treated (L; red) BMDM derived from male and female, wildtype (WT) and IRAP knockout (KO) mice. Data is presented relative to protein concentration and the mean optical density (OD) of the male WT control and was analyzed using a three-way ANOVA with Tukey’s post-hoc test, *p<0.05, n=3 (3 samples per group on separate blots). **(C)** Representative immunofluorescent images and **(D)** semi-quantification of % colocalization of IRAP (green) and CD45 (red) in BMDM from WT mice. Data was analyzed using a two-way ANOVA with Tukey’s post-hoc test, n=3. All data is presented as mean ± SEM.

To further examine the physiological role of IRAP in macrophages, the expression and distribution of the enzyme was examined in BMDM. LPS treatment induced a 3-4-fold increase in expression of IRAP protein in BMDM lysates from both male and female WT mice (**Figures 9A, B**). This increase in IRAP expression with LPS is supported by previous findings in mouse peritoneal macrophages (29). Immunostaining of IRAP in CD45^+^ BMDM revealed that the enzyme is located at the plasma membrane as well as in endosomal compartments. However, the changes in the subcellular distribution of IRAP in response to LPS stimulation is difficult to deduce as the activation of these BMDM resulted in an increase in cell size and shape (**Figure 9C**). Across all groups, there was only 12.85 ± 0.85% colocalization of IRAP and CD45, validating that IRAP is not highly expressed at the plasma membrane but largely remains confined to endocytic vesicles (**Figure 9D**). This semi-quantitative result supports previous observations that ~10% of IRAP is at the cell surface in a basal state at a given point in time (9).

### TLR4 is highly expressed at the cell surface in male IRAP deficient macrophages

The N-terminal domain of IRAP has been found to regulate the trafficking of specialized endosomes containing pro-inflammatory cargo in DCs (7), consequently restricting downstream pro-inflammatory pathways. It is possible a similar mechanism exists in macrophages, whereby IRAP may regulate the trafficking of components associated with a pro-inflammatory response such as the LPS receptor, TLR4, and thus control the extent of its downstream signaling.

**Figure 10:**
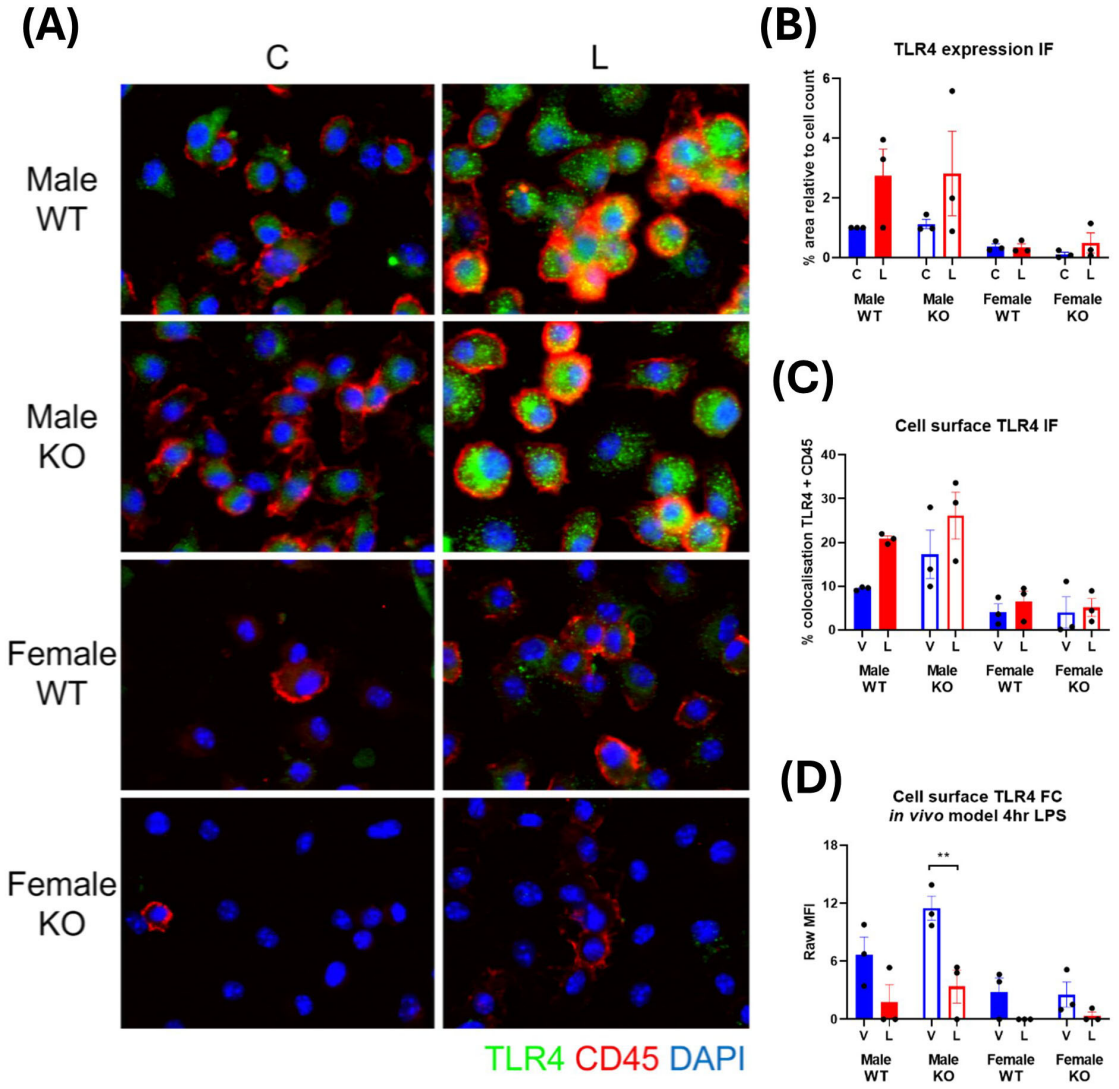
TLR4 is highly expressed at the cell surface in male IRAP-deficient macrophages. **(A)** Representative immunofluorescent images of TLR4 (green) in untreated control **(C)** and LPS-treated (L) CD45^+^ (red) BMDM derived from male and female wildtype (WT) and IRAP knockout (KO) mice. **(B)** Semi-quantification of TLR4 expression from immunofluorescent (IF) images of BMDM measured as % area positive staining relative to the cell count. **(C)** Semi-quantification of the TLR4 expressed at the cell surface of BMDM in IF images measured as % colocalization of TLR4 and CD45. **(D)** Raw mean fluorescence intensity (MFI) of cell surface TLR4 expression in CD45^+^ F4/80^+^ CD206^-^ M1 macrophages in the spleens of mice treated for 4 hours with vehicle control (V) or LPS (L) measured via flow cytometry. All data was analyzed using three-way ANOVAs with Sidak’s post-hoc test, **p<0.01, n=3. All data is presented as mean ± SEM.

Immunofluorescent staining revealed TLR4 is not highly expressed in basal CD45^+^ BMDM and has a dispersed cytosolic distribution (**Figure 10A**). A significant change in TLR4 expression occurred with LPS stimulation with ~3-fold increases in male-derived cells and only very minor, inconsistent increases in female-derived cells (**Figure 10B**). This supports previous literature which demonstrate increases in TLR4 expression with LPS stimulation (30, 31). Surprisingly, there was also a significant sex effect with females expressing lower levels of TLR4 than males both at baseline and after LPS challenge (p=0.002, three-way ANOVA). There was no effect of genotype on TLR4 expression. This suggests that IRAP does not influence the expression of TLR4 at steady state or in response to LPS.

IRAP appeared to influence the subcellular distribution of TLR4 in a basal state in male-derived macrophages where comparison of the percentage of TLR4 colocalized with the cell surface marker, CD45, revealed that cells derived from male IRAP KO mice appear to have a higher basal cell surface level of TLR4 (17.28 ± 5.49%) compared to cells derived from WT mice (9.45 ± 0.24%; **Figure 10C**). This result was recapitulated *in vivo*, with higher basal cell surface levels of TLR4 in CD45^+^ F4/80^+^ CD206^-^ M1 macrophages in the spleen of male IRAP KO mice (11.50 ± 1.25 MFI/geometric mean of expression) compared with male WT mice (6.66 ± 1.83 MFI/geometric mean of expression), measured via flow cytometry (**Figure 10D**). In contrast, there was a similar cell surface expression of TLR4 in steady state cells from female mice across genotypes. This difference in the cellular localization of TLR4 in a basal state that may be regulated by IRAP could dictate the responsiveness of the cell to LPS.

## Discussion

Evidence in support of a role for IRAP in mediating an acute systemic inflammatory response include the high expression of IRAP in immune cells including DCs (7), macrophages (8), mast cells (9) and T cells (10), and that the global gene deletion of the enzyme was found to promote an exaggerated pro-inflammatory response in DCs (7). These immune functions appear clinically relevant given a genetic variant of IRAP, involving a SNP (rs18059), was found to be associated with an increase in 28-day mortality in patients with septic shock, although sex was not a confounding factor in this association (6). The endotoxin, LPS, has been used to induce a systemic inflammatory response that mimics some of the early clinical features of gram-negative sepsis. Therefore, the use of LPS as a preclinical model to investigate the potential role for IRAP in the complex dysregulation of inflammation associated with sepsis is justifiable. This study concurs with previous reports that a heightened response to an immune challenge was observed in DCs from IRAP KO mice, although this was only seen in DCs derived from female mice. In contrast, IRAP gene deletion caused a modest suppression of the CD40 activation marker in M2 macrophages in male but not female mice. This is the first report of an IRAP-linked sex difference in the responsiveness of immune cells to induction of acute inflammation by systemic LPS.

Although no significant genotype differences were observed in terms of body weight or spleen weight in the *in vivo* LPS model, changes in the proportion and activation state of immune cell populations in the spleen still have significant physiological implications. Following detection of LPS in the circulation, there is rapid mobilization of innate myeloid cells such as DCs and monocytes away from bone marrow to immune organs and tissues such as the spleen (<4 hours). In the spleen, there is maturation of these myeloid cells leading to the secretion of cytokines and chemokines and the subsequent activation of nearby white pulp T cells and B cells (12-24 hours). The resulting pro-inflammatory cascade has profound systemic effects in organs such as the heart and lungs, highlighting the widespread implications of changes to immune cell activation in the spleen. This typical response to LPS administration is mirrored in our study whereby mature myeloid cell populations are maximally activated at the 24-hour timepoint. This may contribute to the observations of IRAP-related changes at this timepoint where LPS has its greatest detectable effects.

Given the complex, dynamic and time-sensitive nature of the pro-inflammatory response to systemic LPS, the role of IRAP in LPS-induced inflammation was investigated further in macrophages under controlled conditions in an *in vitro* BMDM model. However, it is worth noting that while both splenic macrophages and BMDM share similar functional properties such as their antigen presenting capacity, there is significant phenotypic and functional heterogeneity (32, 33), likely due to the different macrophage subpopulations present in the spleen such as red pulp macrophages, white pulp macrophages, CD169^+^ marginal zone macrophages and SIGN-R1 CD209b^+^ marginal zone macrophages (34). Furthermore, BMDM and splenic macrophage subpopulations can be at varying levels of maturation (32). Therefore, it is not unexpected that a regulatory role of IRAP was more readily detectable in a single homogenous macrophage population in an early stage of maturation in the BMDM model.

Two reports to date have explored the function of IRAP in macrophages (8, 29). Importantly, whether the role of IRAP in macrophages transitions from physiological to pathological depends on the context, with age and sex being important contributing factors. Drajac, Laubreton (8) highlights how age can influence whether IRAP exerts beneficial or detrimental effects on the immune response. Since neonates are unable to mount an appropriate immune response to RSV infection, the hyper-responsiveness of IRAP deficient macrophages in their study improves survival of the mice (8), suggesting that the role of IRAP in suppressing pro-inflammatory pathways has become detrimental in this context. Observations in splenic macrophages in the current study support their findings in adult mice.

This is the first observation of a sex-dependent difference in the responsiveness of mice with IRAP gene deletion to an LPS-induced immune response. A previous study on bone marrow-derived DCs reported that IRAP gene deletion did not affect the production of pro-inflammatory cytokines 16 hours after LPS stimulation (7). However, the sex of the animals where the DCs originated from was not disclosed (7). Most investigations on the role of IRAP in immune cells have focused predominantly on males or have not specified the sex of the animals (7, 8, 29). A sex difference in IRAP expression has been reported in rat spinal cord where the enzyme was found to be 2.5 times higher in females than in males (35). The authors concluded that this increased IRAP expression may contribute to enhanced oxytocin degradation leading to the suppression of its anti-hyperalgesia effect in female rats (35).

Sex related differences have also been reported in immune responses to infection where, in a number of species, females are observed to mount stronger innate and adaptive immune response than their male counterparts (36). The differential expression and regulation of pattern recognition receptors like TLR4, has a significant impact on sex related differences with androgen and estrogen response elements present in the promoter regions of several innate immunity genes (36). For example, macrophages isolated from the peritoneal cavity of males are more responsive to LPS stimulation compared to that of females, likely due to the increased expression of TLR4 in male-derived macrophages (37). Our findings in BMDM concur with this observation of increased TLR4 expression in male-derived macrophages. These sex-related differences in the immune response highlight limitations in published studies which investigate immunological responses only in male mice.

Previous studies examining the function of IRAP in DCs and T cells have provided leads on potential mechanisms underlying the role of IRAP in macrophages. The cytosolic N-terminal domain of IRAP contains motifs that regulate the retention and trafficking of intracellular endosomal compartments as dysregulated vesicular location and trafficking were observed following global IRAP gene deletion but not in cells expressing a catalytically inactive mutant nor after IRAP inhibitor treatment (7, 10, 38). In DCs or T cells, this domain of IRAP was found to regulate the trafficking of specialized endosomes or phagosomes containing pro-inflammatory cargo such as TLR9 or the CD3ζ chain, consequently restricting downstream pro-inflammatory pathways such as TLR9 signaling (7, 10, 38). Moreover, in DCs, IRAP-containing vesicles were tethered to the actin cytoskeleton via interaction of this N-terminal domain with FHOD4 (7). Given this tethering role of IRAP has been consistently reported across multiple cell types including via FHOD4 in DCs (7) and via AS160 in adipocytes (39), it is conceivable that a similar mechanism exists in macrophages whereby IRAP stabilizes vesicles containing pro-inflammatory cargo such as TLR4 thus dampening the pro-inflammatory response. Therefore, the absence of IRAP would result in increased trafficking of these vesicles and a heightened inflammatory response. It is worth noting that Babdor, Descamps (7) reported no changes in cytokine secretion following TLR4-activation with LPS in bone marrow-derived DCs from WT compared with IRAP KO mice, although differences in TLR4 trafficking and signaling between macrophages and DCs have been proposed (40). As the LPS receptor, TLR4 plays a key role in M1 macrophage activation and has a well-established trafficking route (40) that may partially overlap with that of IRAP. In agreement with other published studies (36), our findings suggest that male IRAP KO mice have higher basal cell surface levels of TLR4 than their WT counterparts. This may account for the heightened sensitivity of these macrophages to LPS stimulation at the plasma membrane.

In summary, we have identified a sex-dependent role of IRAP in regulating LPS-mediated dendritic cell and macrophage activation. Given LPS can mediate gram-negative sepsis and its plasma levels correlate with sepsis severity and mortality (41), the identification of IRAP as a potential regulator of LPS responsiveness advances our understanding of the mechanisms underlying the acute pro-inflammatory phase of gram-negative sepsis in humans. This potential involvement of IRAP in septic shock was supported by the clinical report that genetic variations in the regulatory region of the LNPEP gene (SNP rs4869317, TT genotype) are significantly associated with increased mortality (as the primary outcome variable), as well as increased AVP clearance and serum sodium levels (as secondary outcome variables) (6). The authors of the study hypothesized that the increased mortality was linked to greater AVP clearance possibly due to the upregulation of IRAP activity. Our findings suggest an alternative or additional interpretation of the clinical observation and highlight a sex-dependent role for IRAP in acute inflammatory responses in sepsis.

## Data Availability

The original contributions presented in the study are included in the article/**Supplementary Material**. Further inquiries can be directed to the corresponding author.
