# Supplementary material for: Sex- and time-dependent role of insulin regulated aminopeptidase in lipopolysaccharide-induced inflammation
Source: Front Immunol. 2024 Oct 4;15:1466692. doi: 10.3389/fimmu.2024.1466692 (PMC11486674; doi:10.3389/fimmu.2024.1466692)
Supplement: Supplementary file 1 [file DataSheet1.docx]

**Supplementary Information**

***Supplementary Table 1. Summary of all data from the in vivo LPS study.*** *Flow cytometry results derived from two-way ANOVAs showing treatment effects (vehicle vs LPS), genotype effects (wildtype vs IRAP KO) or interactions on the frequency of the cell population and expression of the activation markers CD40, CD80, CD86 and MHCII in dendritic cells (DCs), M1 and M2 macrophages, T helper cells and B cells in the spleen of male or female mice (aged 10-15 weeks) at 4, 24 or 48 hours post-LPS injection. Only significant results from the two-way ANOVAs are shown *p<0.05, **p<0.01, ***p<0.001, ****p<0.0001. Specific post-hoc analyses are discussed in the results text. ns = non-significant, NA = not applicable as no data was obtained due to lack of positive signal.*

| **Population** | **Time point** | **Parameter** | **Male p values** | **Female p values** |
| --- | --- | --- | --- | --- |
| DCs | 4hr | Frequency | ns | Genotype = 0.0110* |
|  |  | CD40 | ns | ns |
|  |  | CD80 | ns | ns |
|  |  | CD86 | Treatment = 0.0133* | Treatment = 0.0159* |
|  |  | MHCII | ns | Treatment = 0.0500* |
|  | 24hr | Frequency | Treatment < 0.0001**** | Treatment < 0.0006*** |
|  |  | CD40 | Treatment < 0.0001**** | Treatment < 0.0001****  Genotype = 0.0195*  Interaction = 0.0195* |
|  |  | CD80 | Treatment = 0.0050** | Treatment = 0.0144* |
|  |  | CD86 | Treatment < 0.0001**** | Treatment < 0.0001****  Genotype = 0.0396*  Interaction = 0.0396* |
|  |  | MHCII | ns | Treatment = 0.0002***  Genotype = 0.0315*  Interaction = 0.0315* |
|  | 48hr | Frequency | ns | Treatment = 0.0362* |
|  |  | CD40 | Treatment = 0.0125* | Treatment = 0.0004*** |
|  |  | CD80 | ns | ns |
|  |  | CD86 | ns | ns |
|  |  | MHCII | Treatment < 0.0001**** | Treatment < 0.0001**** |
| M1 macrophages | 4hr | Frequency | ns | ns |
|  |  | CD40 | Treatment < 0.0001**** | Treatment = 0.0008*** |
|  |  | CD80 | NA | ns |
|  |  | CD86 | Treatment < 0.0001**** | Treatment = 0.0013** |
|  |  | MHCII | Treatment = 0.0182* | ns |
|  | 24hr | Frequency | Treatment = 0.0195* | ns |
|  |  | CD40 | Treatment = 0.0005*** | ns |
|  |  | CD80 | ns | ns |
|  |  | CD86 | Treatment = 0.0006*** | ns |
|  |  | MHCII | Treatment = 0.0128* | ns |
|  | 48hr | Frequency | Treatment = 0.0020** | Treatment < 0.0001**** |
|  |  | CD40 | Treatment = 0.0009*** | Treatment < 0.0001**** |
|  |  | CD80 | ns | ns |
|  |  | CD86 | ns | NA |
|  |  | MHCII | Treatment < 0.0001**** | Treatment < 0.0001**** |
| M2 macrophages | 4hr | Frequency | ns | ns |
|  |  | CD40 | Genotype = 0.0130*  Interaction = 0.0130* | ns |
|  |  | CD80 | Treatment = 0.0482* | ns |
|  |  | CD86 | ns | Treatment = 0.0350* |
|  |  | MHCII | ns | ns |
|  | 24hr | Frequency | Treatment = 0.0012** | Treatment = 0.0003*** |
|  |  | CD40 | Treatment < 0.0001****  Genotype = 0.0475*  Interaction = 0.0475* | Treatment = 0.0065** |
|  |  | CD80 | ns | Treatment = 0.0199*  Genotype = 0.0252*  Interaction = 0.0252* |
|  |  | CD86 | Treatment = 0.0001*** | Treatment = 0.0323* |
|  |  | MHCII | ns | ns |
|  | 48hr | Frequency | Treatment < 0.0001****  Genotype = 0.0474* | Treatment < 0.0001**** |
|  |  | CD40 | Treatment = 0.0229* | Treatment = 0.0229* |
|  |  | CD80 | Treatment = 0.0122* | Treatment = 0.0122* |
|  |  | CD86 | ns | NA |
|  |  | MHCII | Treatment = 0.0017** | Treatment = 0.0017** |
| T helper cells | 4hr | Frequency | ns | Genotype = 0.0156* |
|  | 24hr | Frequency | Treatment = 0.0294*  ns | Treatment = 0.0005***  Genotype = 0.0044** |
|  | 48hr | Frequency | Treatment = 0.0001***  ns | Treatment < 0.0001****  Genotype = 0.0291*  Interaction = 0.0100* |
| B cells | 4hr | Frequency | ns | Genotype = 0.0477* |
|  | 24hr | Frequency | ns | ns |
|  | 48hr | Frequency | Treatment < 0.0001**** | Treatment < 0.0001**** |


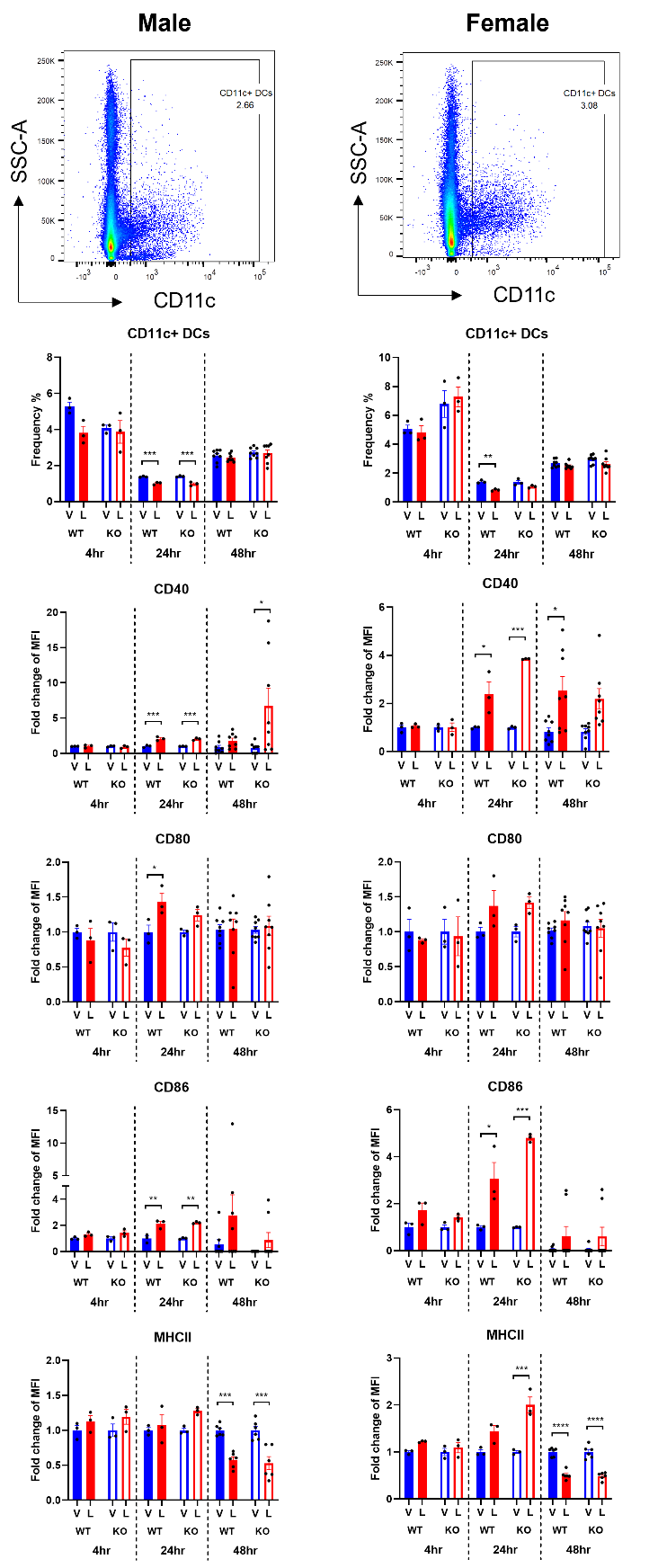


**(A)**

**(B)**

**(C)**

***Supplementary Figure 1. IRAP influences the activation of DCs in the spleen of female mice in response to LPS.*** *(A) Representative flow cytometry dot plot showing the gating for CD11c^+^ DCs in the spleen. (B) The frequency (%) of CD11c^+^ DCs in the spleen of wildtype (WT; filled bars) and IRAP knockout (KO; empty bars) mice (aged 10-15 weeks) administered either vehicle (V; blue) or LPS (L; red) once for 4 hours (n=3) or 24 hours (n=3) or twice over 48 hours (n=6-8). (C) Quantification of the fold changes in mean fluorescence intensity (MFI) compared to the mean of vehicle controls of the activation markers CD40, CD80, CD86 and MHCII in DCs. Data from each timepoint was analysed separately using a two-way ANOVA with Tukey’s post-hoc test, *p<0.05, **p<0.01, ***p<0.001, ****p<0.0001. All data is presented as mean ± SEM.*


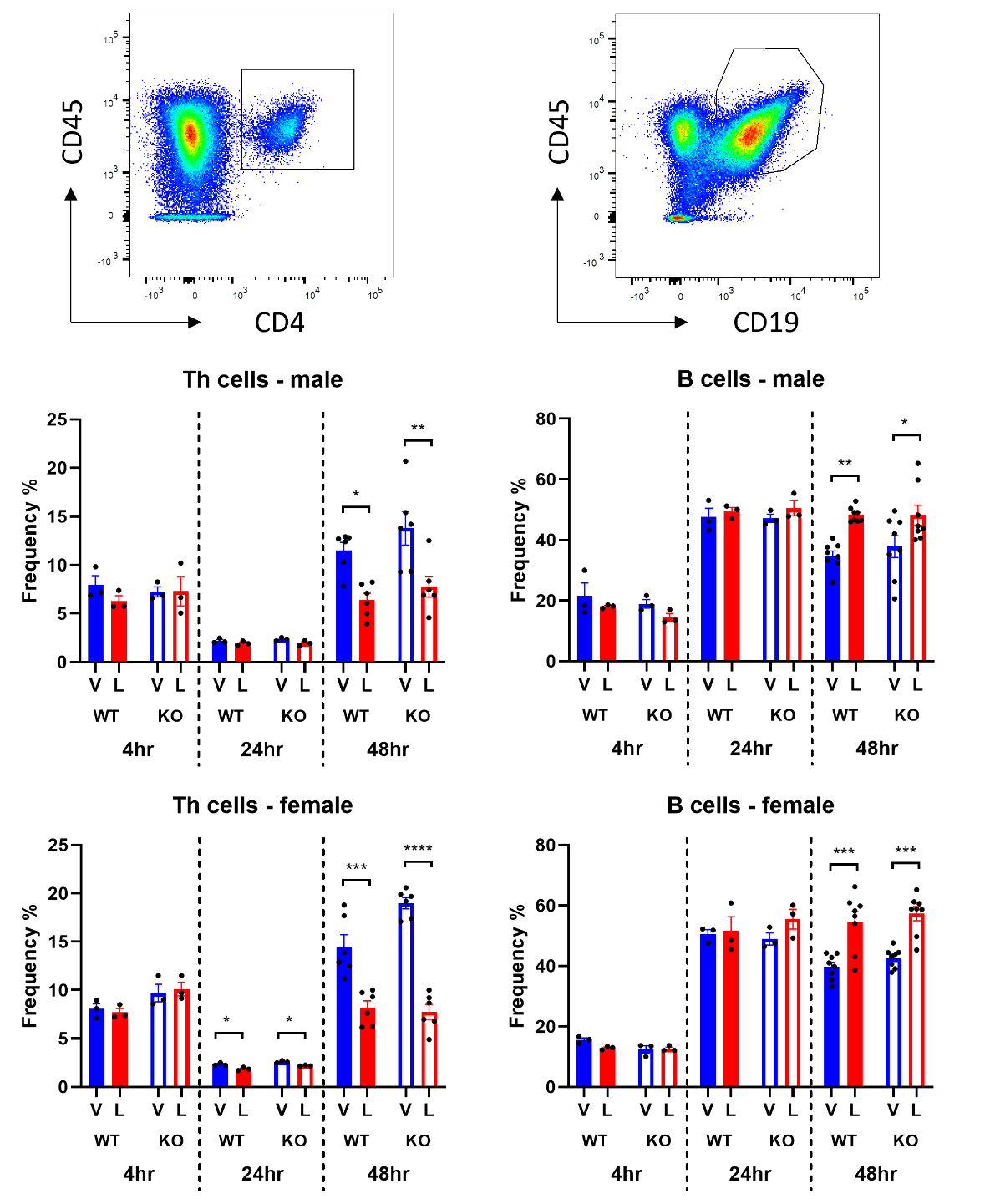


**(A)**

**(B)**

**(C)**

***Supplementary Figure 2. IRAP does not influence the frequency of T helper or B cells in the spleen in response to LPS.*** *(A) Representative flow cytometry dot plots showing the gating for CD45^+^ CD4^+^ Th cells and CD45^+^ CD19^+^ B cells in the spleen of male control mice. The frequency (%) of Th cells and B cells in the spleen of (B) male and (C) female wildtype (WT; filled bars) and IRAP knockout (KO; empty bars) mice (aged 10-15 weeks) administered either vehicle (V; blue) or LPS (L; red) once for 4 hours (n=3) or 24 hours (n=3) or twice over 48 hours (n=6-8). Data from each timepoint was analysed separately using a two-way ANOVA with Tukey’s post-hoc test, *p<0.05, **p<0.01, ***p<0.001, ****p<0.0001. All data is presented as mean ± SEM.*


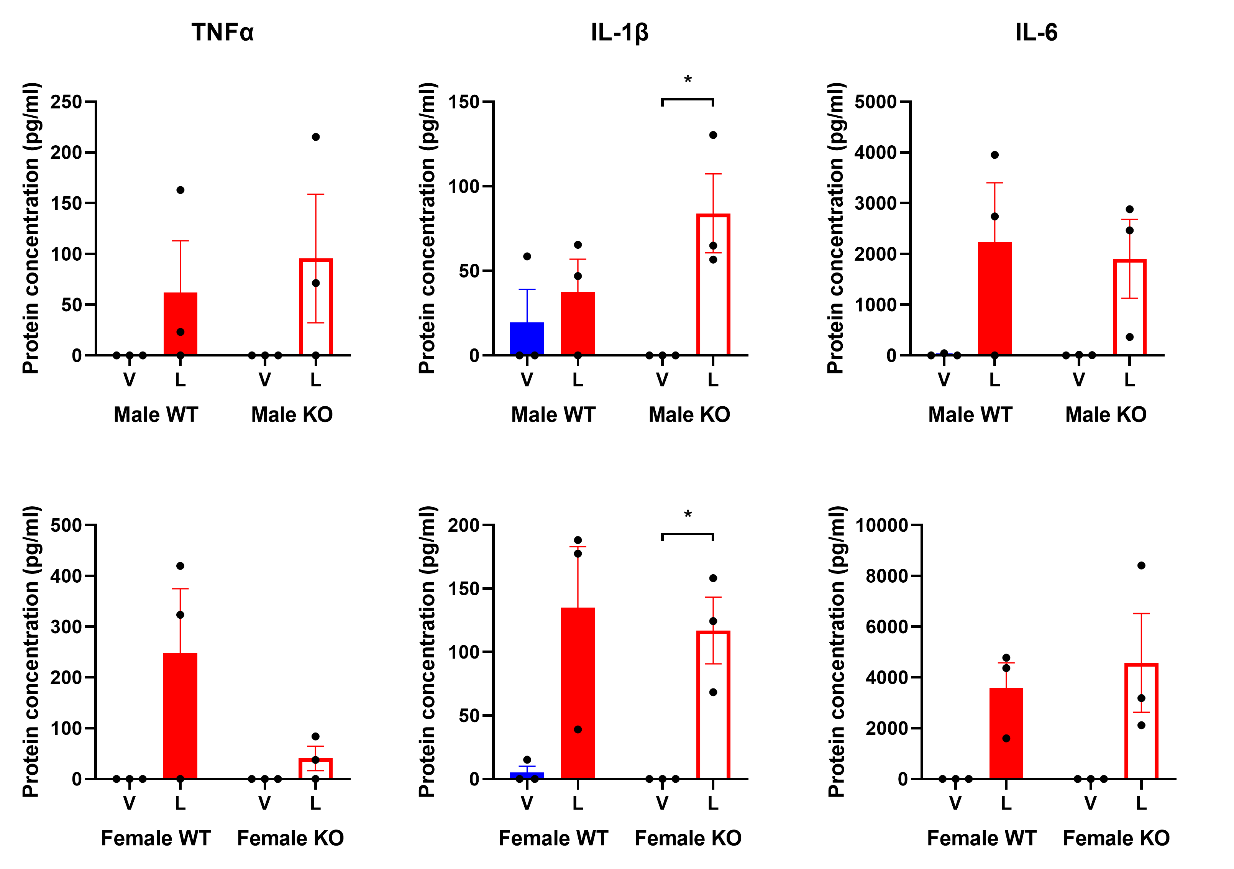


**(A)**

**(B)**

***Supplementary Figure 3. Enhanced secretion of IL-1β in male IRAP KO mice treated with LPS for 4 hours.*** *Protein concentrations (pg/ml) of the pro-inflammatory cytokines TNFα, IL-1β and IL-6 in plasma of (A) male and (B) female wildtype (WT; filled bars) and IRAP knockout (KO; empty bars) mice (aged 10-15 weeks) administered either vehicle (V; blue) or LPS (L; red) for 4 hours (n=3), measured via sandwich ELISAs. Data is presented as mean ± SEM and was analysed using a two-way ANOVA with Tukey’s post-hoc test, *p<0.05 (n=3).*


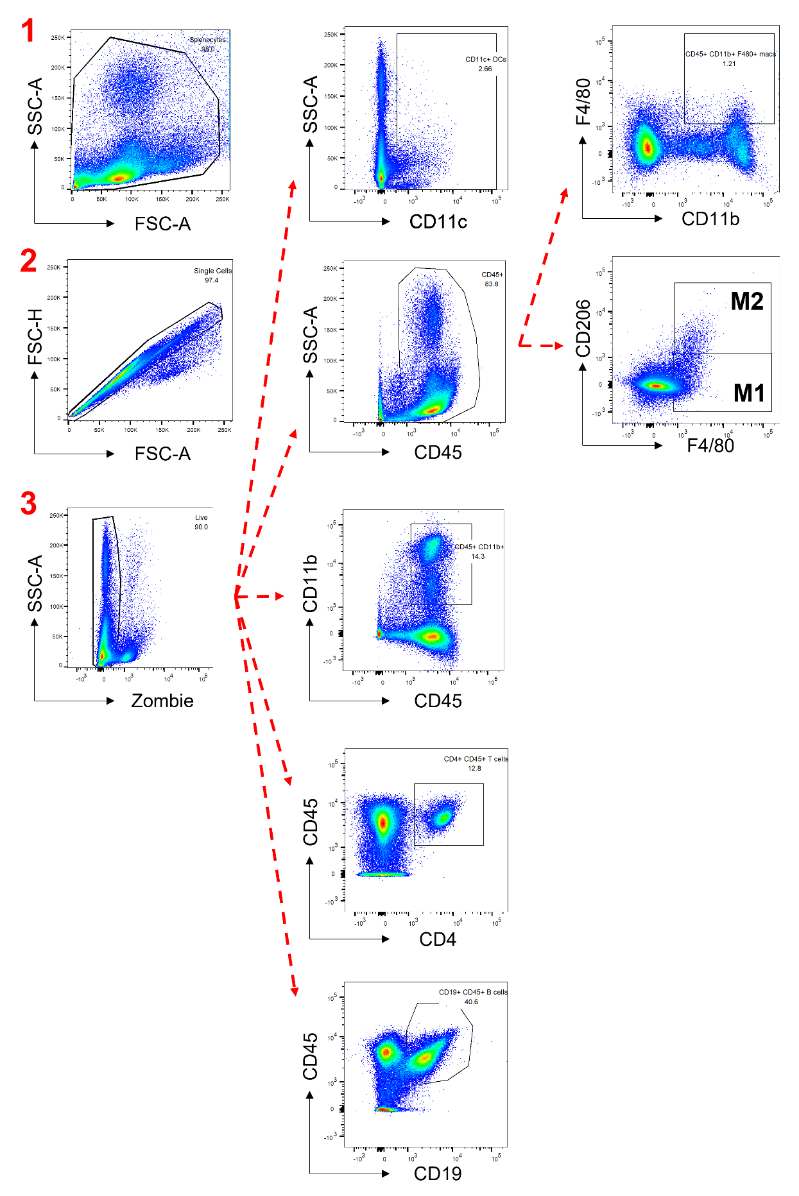

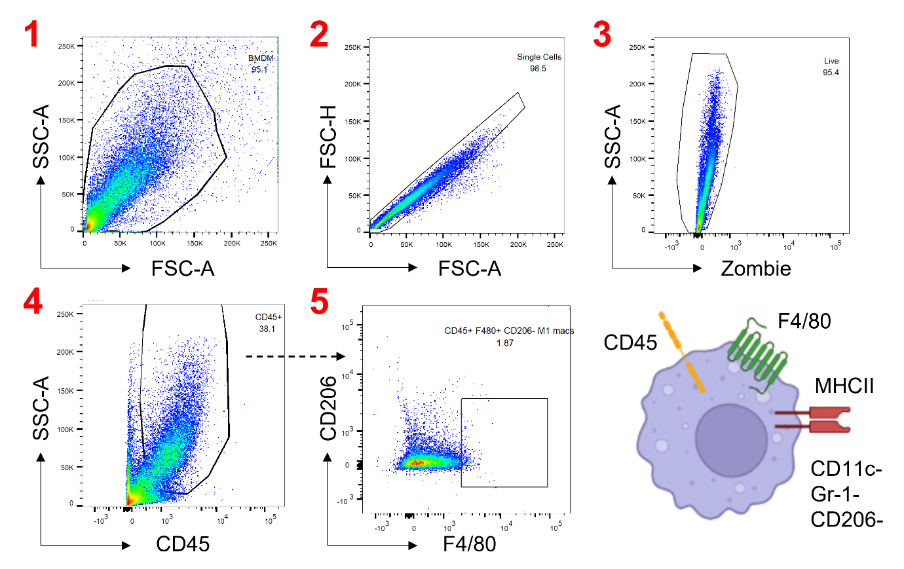


**(A)**

**(B)**

***Supplementary Figure 4. Gating strategy for different immune cell populations in the spleen and in BMDM.*** *(A) In the in vivo LPS model, from the live cell population (3), different immune cell types were identified such as CD11c^+^ dendritic cells, CD45^+^ CD11b^+^ F4/80^+^ macrophages, CD4^+^ CD45^+^ T helper cells and CD19^+^ CD45^+^ B cells. (B) In the in vitro BMDM model, from the live cell population (3), CD45^+^ F4/80^+^ CD206^-^ M1 macrophages could be identified (5).*
